# Study protocol for a randomized controlled trial to test for preventive effects of diabetic foot ulceration by telemedicine that includes sensor-equipped insoles combined with photo documentation

**DOI:** 10.1186/s13063-019-3623-x

**Published:** 2019-08-22

**Authors:** Antao Ming, Isabell Walter, Ahmad Alhajjar, Martin Leuckert, Peter R. Mertens

**Affiliations:** Clinic for Nephrology and Hypertension, Diabetes and Endocrinology, Otto-von-Guericke University, Leipziger Str. 44, 39120 Magdeburg, Germany

**Keywords:** Diabetic foot syndrome, Prevention of DFUs, Temperature measurement, Smartphone application, Sensor-equipped insole

## Abstract

**Background:**

Early detection of diabetic foot ulcerations (DFUs) can avoid or delay any progression into more severe stages, which may require limb amputation or lead to infectious sequelae and death. However, frequent clinical screening would be too intrusive and costly, and self-examination may be hampered by concomitant diseases and social disabilities. In addition, it requires professional knowledge and experience using specialized devices. Researchers reported that skin temperature monitoring could reduce the risk of DFUs in high-risk patients. The main research objects in this field are effective and convenient means of temperature measurement, accurate and reasonable early warning mechanisms, and timely and appropriate interventions. This trial aims to investigate the effectiveness of daily home-based foot temperature measurements in the prevention of DFUs with the aid of intelligent sensor-equipped insoles combined with photo documentation.

**Methods/Design:**

In this open-label, prospective, randomized, 24-month trial, 300 patients with diabetes mellitus (type 1 or 2) and severe diabetic peripheral neuropathy (vibration sensation ≤ 4/8), aged 18–85 years, will be recruited and assigned to control and intervention groups in a ratio of 1:1. Main inclusion criteria to be eligible for study participation encompass in particular risk group 2 or 3 for the development of DFUs using the diabetic foot risk classification system (as specified by the International Working Group on the Diabetic Feet [IWGDF]) and the ability to use a mobile phone.

**Interventions:**

Participants in both groups will receive education about regular foot care at the beginning of the study (visit 0). In the intervention group, every patient will receive a pair of slippers with the inserted sensor-equipped insole as well as a smartphone with the corresponding smartphone application (Smart Prevent Diabetic Feet Application). The insole is a tool that records the temperature variabilities of the plantar foot. Patients will measure their foot temperature twice a day at home with a time interval > 4 h during the entire course of the study (24 months). The measured data will be initially analyzed and visualized, and further transferred to a remote server that allows the physician to perform specific interpretations. In case of temperature differences > 1.5 °C between left and right corresponding sites lasting > 32 h (assigned alarm level 4), the physician will start an intervention phase, which requires the patient to reduce daily activities and relax his feet for five days. At the same time, photo documentation is encouraged to be performed by the patient. Possibly, additional visits to a private doctor or clinical examinations will be arranged for the patient during this intervention period. Outcomes: The primary outcome is foot ulceration, evaluated by a physician, and occurring at any point during the study.

**Discussion:**

This study addresses principal aspects in the prevention of DFUs. First, the sensor-equipped insole will be evaluated for daily performance in home-based measurements of foot temperatures. Second, a telemedicine structure is tested that evaluates sensor data automatically and proposes suitable intervention measures under the supervision of a physician. Third, predictive models for DFUs will be built using the collected sensor data allowing for interpretations, which in the future may support medical care providers.

**Trial registration:**

German Clinical Trials Register (DRKS), DRKS00013798. Registered on 18 January 2018.

**Electronic supplementary material:**

The online version of this article (10.1186/s13063-019-3623-x) contains supplementary material, which is available to authorized users.

## Background

According to the definition of the World Health Organization (WHO), diabetic foot syndrome (DFS) encompasses all foot complications, constituting an “ulceration of the foot (distally from the ankle and including the ankle) associated with neuropathy and different grades of ischemia and infection” [1]. It increases the risk of limb amputation, and even mortality, if left untreated [2]. In Germany, about 40,000 legs, feet, or toes are amputated, with 70% of major amputations and 85% of minor amputations due to DFS. In addition, foot lesions in diabetic patients impose an enormous social and economic burden across the world. In the US, Rogers et al. reported that $18 billion were spent on the care of diabetic foot ulcers (DFUs) and $11.7 billion sum up as consequences of lower extremity amputations [3].

Among the reasons for DFS, diabetic foot neuropathy is the major contributing factor for foot complications (50% as a single cause, 30–50% as a cause in combination with angiopathy [4]), because it affects the ability of the foot to feel and sense [5–7]. This is why patients with diabetic neuropathy are not able to realize injuries to their feet. Most of the complications develop due to infection and ulceration in the foot [8, 9]. The early signs of DFS include fissures, blisters, abundant callus formation, redness, and increased temperature [10]. A physician may diagnose the exact cause by analyzing these physical features [11].

It is possible to delay or even avoid the development of DFUs with adequate treatment at early stages. Usually, clinicians assess the general condition through analyzing ankle brachial pressure indices, plantar pressure profiles, and testing for foot neuropathy [12]. Additionally, advanced technologies like corneal confocal microscopy, magnetic resonance tomography, and Doppler ultrasonography provide tools to diagnose the prevalence of peripheral neuropathy and angiopathy, foot ulcers, and its risks [13]. However, these methods are considered intrusive and are costly; patient compliance is lacking, especially with frequent doctor’s visits [2]. On the other hand, patient self-assessment has limitations such as lack of knowledge about this condition, difficulties using specialized equipment, and impaired physical mobility. More effective and advanced approaches need to be investigated to provide flexible and comprehensive foot care for patients at risk for the DFS.

Elevated plantar temperatures have been reported to be an early sign of incipient DFUs. In the studies of Lavery et al. and Armstrong et al., home temperature monitoring and reduced activities have been verified to be effective to reduce the incidence of DFUs in high-risk patients [14, 15]. In the study of Lazo-Porras et al., the effectiveness of foot thermometry (TempStat™ for thermal image capture) to prevent DFUs was investigated, together with mHealth reminders (SMS and voice messaging), in an evaluator-blinded randomized 12-month trial. The authors highlighted the importance to evaluate adherence to daily home-based measurements [16]. Furthermore, a left-to-right foot temperature difference of > 2.2 °C as a proposed threshold for an impending ulceration has been investigated comprehensively by Wijlens et al. in 20 patients with diabetes and peripheral neuropathy. Their conclusion was that the > 2.2 °C threshold is only acceptable if it is confirmed after 24 h in a repeated measure and if, in addition, the temperature difference is individually corrected depending on baseline measurements [17]. In addition to neuropathic ulcers [18, 19], one has to consider osteomyelitis [20, 21] and the disease termed Charcot foot [22] as differential diagnoses in the case of elevated plantar temperatures.

On the other hand, decreased foot temperatures may point to a vascular insufficiency in the foot [23]. Therefore, foot temperature monitoring with thermometers, thermal imaging techniques, wearable temperature techniques (socks, insoles, and shoes) has been widely tested to date. For example, Netten et al. explored the temperature discrimination thresholds between “no,” “local,” or “diffuse” DFUs with a high-resolution infrared thermal imaging technique [24]. Fraiwan et al. implemented a mobile thermal imaging system with an automated method to identify possible ulcers in diabetic patients [11]. These pioneering works may open a window for patients to check for their foot condition in a feasible and comfortable fashion in the future.

Moreover, in the study by Fryberg et al., a novel smart mat technology was evaluated for predicting impending DFUs in a 34-week cohort study that enrolled 132 patients with diabetes. Their results support the notion that the remote temperature-monitoring system could be a feasible and efficient strategy to early identify DFUs, but the asymmetry thresholds have a significant influence on the sensitivity and specificity. Comparing the 2.22 °C and 3.20 °C thresholds, sensitivity decreased from 97% to 70%, but the specificity increased from 43% to 68% [25]. Therefore, effective and convenient means of temperature measurements such as home-based wearable technologies, accurate and reasonable early warning mechanisms with disparate asymmetry thresholds, followed by timely and appropriate interventions are the main research focus in this field.

From our perspective, home-based monitoring of plantar foot temperatures may be regarded as an effective method in the early detection and possible prevention of DFUs. In this study, by utilizing a novel sensor-equipped insole, we aim to establish a telemedicine structure with a remote server and the corresponding smartphone app to timely monitor changes of plantar foot temperatures in diabetes patients. The evidence obtained will include a set-up with predefined standardized temperature recordings and a telemedicine aspect allowing for feedback and alarming as well as picture recordings. The outcome of our study will ultimately allow us to determine if and to which extent such an effort may reduce the number of diabetic foot ulcerations and other medical foot conditions in such a cohort.

## Methods/Design

### Objectives

The present study aims to investigate the hypothesis that a twice-daily recording of foot temperatures with the aid of the sensor-equipped insole (Medixfeet Insole®, Thorsis Technologies GmbH) can reduce the risk of ulcer formation.

### Primary specific aim

The primary objective of the present study was to compare the incidence of DFUs during the study period between patients who only receive education about regular foot care and those patients who additionally proceed with daily measurements of foot temperatures with the sensor-equipped insole, together with an app-based warning system and self-imaging of feet for incipient ulcer development.

### Secondary specific aims

The secondary objectives of the present study were to:

1. collect safety-relevant information concerning the equipment (insole)
  - frequency of adverse events (AEs)
  - frequency of serious adverse events (SAEs)
2. quantify precursors of the primary endpoint
  - redness in the foot area
  - infections in the foot area
  - wounds in the foot area
3. evaluate the changes of quality of life independent of primary and secondary endpoints
4. assess the adherence to daily two-time temperature measurements based on data acquisition by the app
5. record the alarm frequency in the intervention group based on data collected by the app
6. detect “slow” temperature drops as an indicator of circulatory disorders
7. assess the adherence to photo documentation

### Study design

This open-label trial will randomize 300 high-risk patients with diabetes and advanced polyneuropathy, that lack severe peripheral angiopathy, into two groups with a 1:1 ratio.

**Fig. 1:**
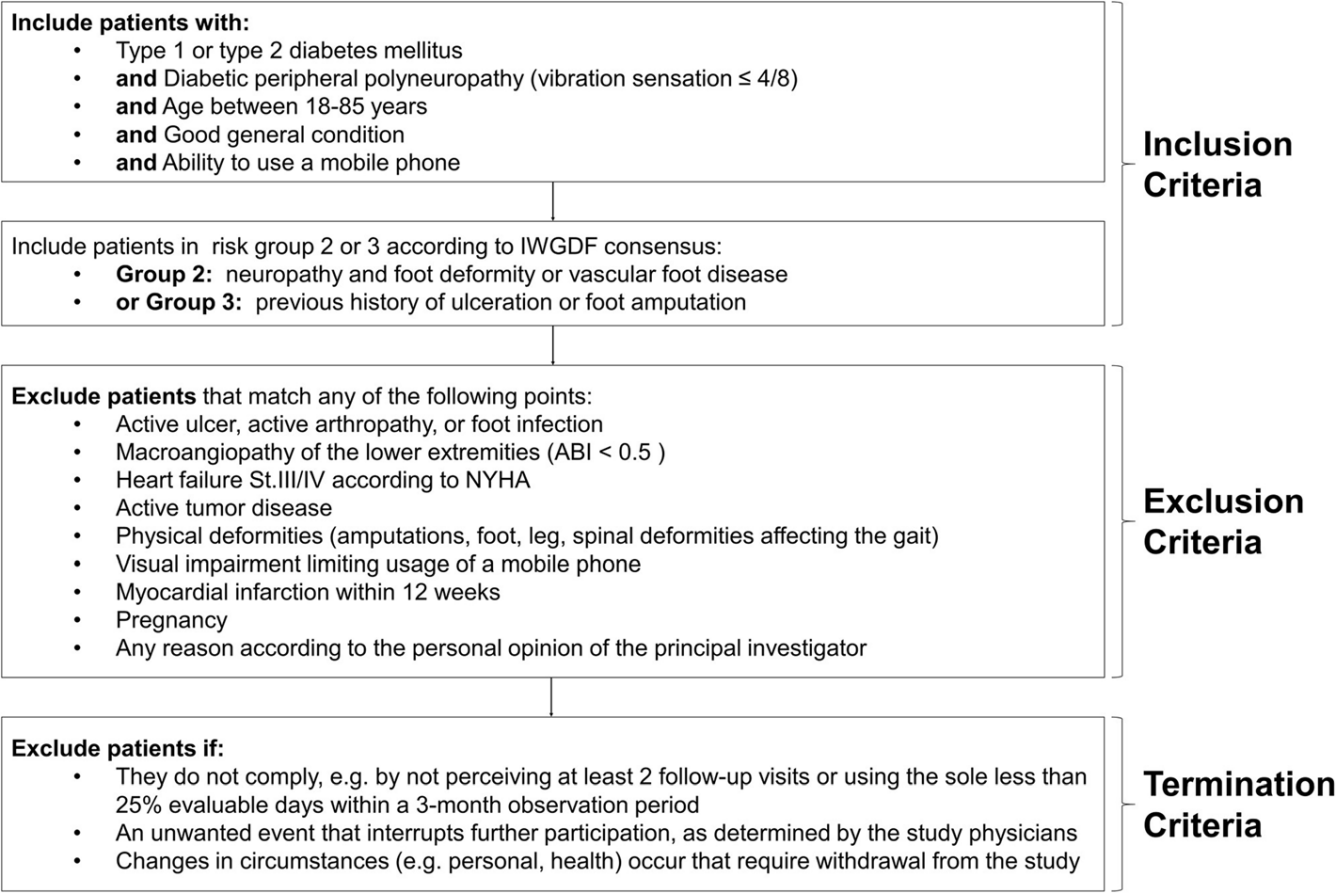
Algorithm for possible study enrollment, with description of the inclusion, exclusion and termination criteria of the study in the screening process

At the screening visit, all potential study participants will first be informed about the aim and purpose of the study. They will be interviewed for past medical foot problems with documentation thereof and will thereafter be examined for polyneuropathy and blood circulation disorders (see below). Regarding the study-specific inclusion and exclusion criteria (compare Fig. 1), the study physician informs the patient about their possible suitability for participation and the modalities of the study. If patients are eligible for the protocol, they will be enrolled only after giving informed consent (see Additional file 1).

**Fig. 2:**
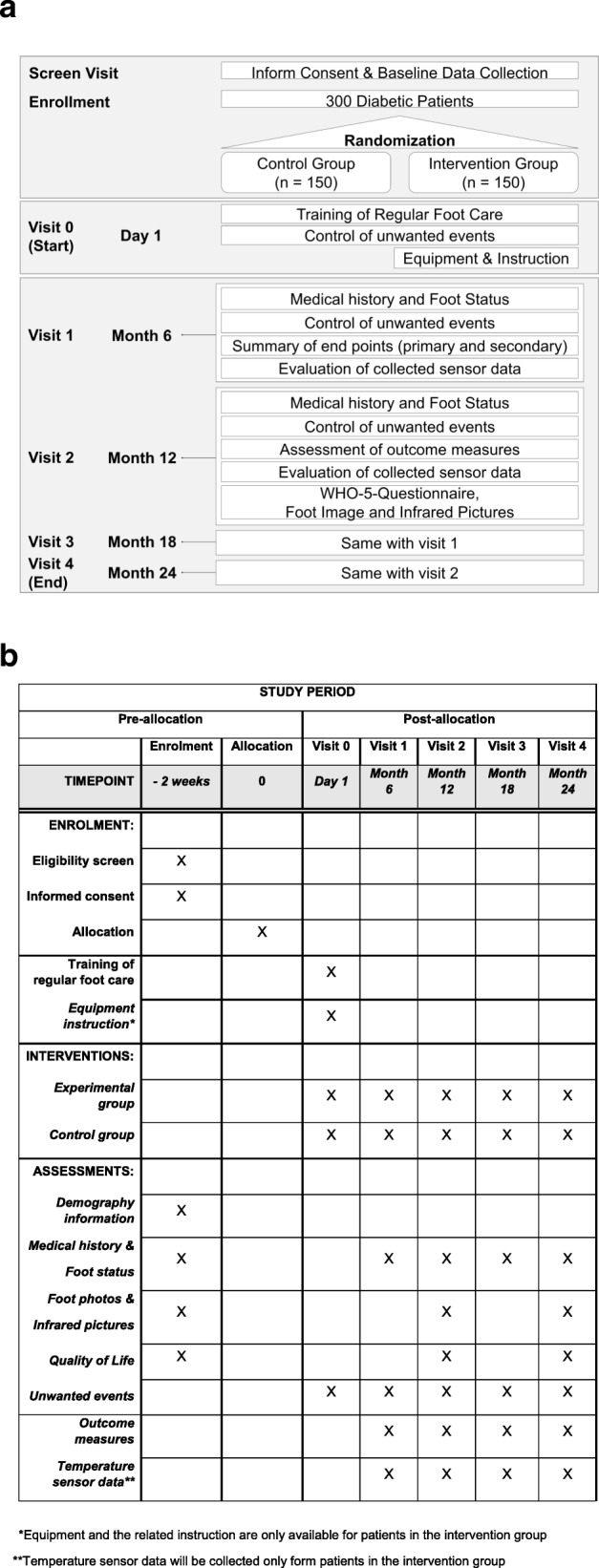
**a*** Flow diagram* of the study showing procedures, activities, and processes. **b*** Figure* showing the Standard Protocol Items: Recommendations for Interventional Trials (SPIRIT see Additional file 4) for this study

**Fig. 3:**
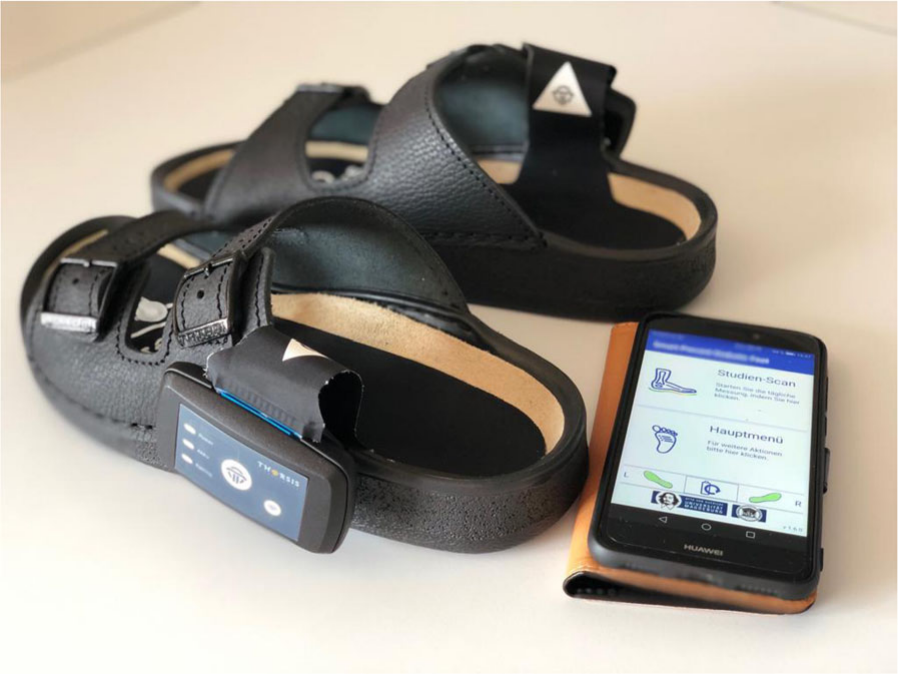
Study materials for patients in the intervention group showing the sensor-equipped insole (Medixfeet Insole®, Thorsis Technologies GmbH) and the Smart Prevent Diabetic Feet Application (SPDFA)

In a next step, patients will be randomized into control or intervention group in a ratio of 1:1. Two weeks later, at visit 0, they will be trained by a qualified study physician about regular foot care measures to prevent foot ulcers (standardized patient education) (see Additional file 2). The non-intervention group will not undergo any further immediate intervention; however, research participants in this group will be seen at regular follow-up visits at six-month intervals (Fig. 2). In the intervention group, every patient receives a pair of slippers with inserted sensor-equipped insoles as well as a smartphone with a Smart Prevent Diabetic Feet Application (SPDFA) (Fig. 3, see Additional file 3). They will perform measurements of their foot temperatures twice a day at home, with time intervals > 4 h during the entire study course, which comprises 24 months. In case of temperature differences > 1.5 °C between left and right corresponding sensor sites, and lasting > 32 h, the study participant will be instructed to reduce daily activities and relax his feet for five days. Additionally, the participant will receive a notification by the app to take pictures with his smartphone from the dorsal and (possibly with help of care providers) plantar feet that are transferred to the study center. In the app, essential guidance and foot masks help the patient to capture standardized foot images (Fig. 5). Depending on the findings of the photo documentation, additional visits to the study center to perform clinical examinations will be arranged for the patient during this intervention period.

In addition, patients in both groups are required to consult a physician when early signs of foot ulceration are noted by self-inspection of the feet (e.g. redness, pain, sores). Follow-up visits are carried out after enrollment in the study at months 6, 12, 18, and 24 by a registered nurse and a physician trained to diagnose and treat DFS. Visits 1 and 3 (after 6 and 18 months, respectively) encompass the evaluation of patient’s foot status, control of unexpected events, summary of endpoints, and assessment of the collected sensor data. Visits 2 and 4 (after 12 and 24 months, respectively) additionally include the assessment of outcome measures, patient’s wellbeing evaluation by WHO-5-questionaire, as well as taking pictures with normal illumination and infrared light from the patient’s feet (Fig. 2).

The primary endpoint of the study is DFU formation (more precisely, the time until ulcers form) and the total number of ulcerations in each group. Secondary endpoints will include evaluation of AEs and SAEs, precursors of the primary endpoint as listed above, assessment of quality of life using an interactive patient’s diary (Fig. 6), patient compliance, information about temperature alarms—including “slow” temperature drops, and acquisition of photo documentation.

If a foot ulcer occurs in a patient, it will be treated according to the usual measures of standard clinical care. Possible discontinuation of the study occurs according to the defined termination criteria (Fig. 1).

The SPDFA receives the measured data of the sensor-equipped insole via low-energy Bluetooth® connectivity. It then performs an initial analysis of temperature differences and visualizes these. Thereafter, the temperature recordings are transferred from the SPDFA to a study server. This server is located in the premises of the computer center of the Medical Faculty of the Otto-von-Guericke University Magdeburg. The data may be exported from the study server in a suitable format (SAS / SPSS) for statistical analysis at the Institute of Biometry and Medical Informatics. The final report will be compiled no later than one year after the end of the study.

### Participant recruitment and selection criteria

Recruitment is carried out by practicing diabetologists and podiatrists in the Polyclinic of the University Hospital Magdeburg. The study will enroll 300 patients aged 18–85 years with type 1 or type 2 diabetes mellitus and exhibiting severe diabetic peripheral neuropathy (vibration sensation ≤ 4/8) with or without a history of ulceration. They will be eligible only when they are classified as high-risk patients, i.e. risk class 2 or 3 as defined by the diabetic foot risk classification system (as specified by the IWGDF) [26, 27]. It is based on a short questionnaire about previous history of ulceration and/or partial foot amputation, foot evaluation to detect bunion, rigid deformities (such as hammer digit or claw toe), and prominent metatarsal heads, as well as neuropathy testing using the vibration perception threshold and the Semmes-Weinstein monofilament [28]. The participants of the study have to be able to use a smartphone and its applications. The study excludes patients with active ulcer, arthropathy, tumor disease, as well as those with foot infection, macroangiopathy of the lower extremities (ABI < 0.5), heart failure classes III/IV according to NYHA, physical deformities (amputations, foot, leg, spinal deformities affecting the gait), visual impairment that limits normal use of smartphones, myocardial infarction within 12 weeks before study protocol inclusion, or pregnancy. The principal investigator has the right to preclude participation due to any reason in his personal opinion and in accordance with the inclusion and exclusion criteria as summarized (Fig. 1).

### Baseline data collection

At the screening visit, the study physicians record the past medical history by means of a foot documentation sheet recommended by the Foot Working Group of the German Diabetes Society. It includes the following items:

1. Previous foot lesions, deformities, and surgeries
2. Details about the previous shoe supply
3. Presence of blood supply disorders (ischemia and PAD)
4. Burning, numbness, weakness, cramps or pain in the legs and feet

In addition to the interview, a series of tests are carried out to determine the degree of polyneuropathy and blood supply disorders:

1. Monofilament test to check the sensation of touch and pressure
2. Tip-Therm Test to check the temperature sensation
3. Tuning fork test for measuring depth sensitivity and vibration sensation
4. Testing the sensation of pain with a disposable needle
5. Doppler ultrasound test for the measurement of circulatory disorders
6. Muscle self-reflex status
7. Blood pressure measurement (for ABI)

If patients are eligible for the protocol, the study physician obtains informed consent at screening visit through a written consent form with the signature of the potential trial participant.

### Randomization

In the University Clinic for Nephrology and Hypertension, Diabetes and Endocrinology, randomization is performed using the software RITA (from Statsol, Lübeck). Three hundred patients will be assigned to two groups with a 1:1 ratio based on a stratification according to the prevalent risk group (2 or 3), gender, age (< 60 years vs ≥ 60 years), and the degree of neuropathy (restriction of vibration sensation, using the minimization algorithm of Pocock and Simon [29]). Randomization to the study protocol will be based on the intention-to-treat principle. The randomization is not concealed to the physicians and to the study population at any time after informed written consent of the patients.

### Intervention

The sensor-equipped insole (Medixfeet Insole®, Thorsis Technologies GmbH, Magdeburg, Saxony-Anhalt, Germany) features six temperature sensors that measure the foot temperatures at different locations. These are the plantar hallux (D1), the first, third, and fifth metatarsal heads (MTK1, MTK3, and MTK5), the mid-foot (lateral), and the heel (calcaneus). From our previous experience considering both energy and performance aspects, the duration of each single measurement is set at 3 min using a measuring frequency of 2 Hz. The measured temperature data will be transferred via Bluetooth® to a smartphone.

**Fig. 4:**
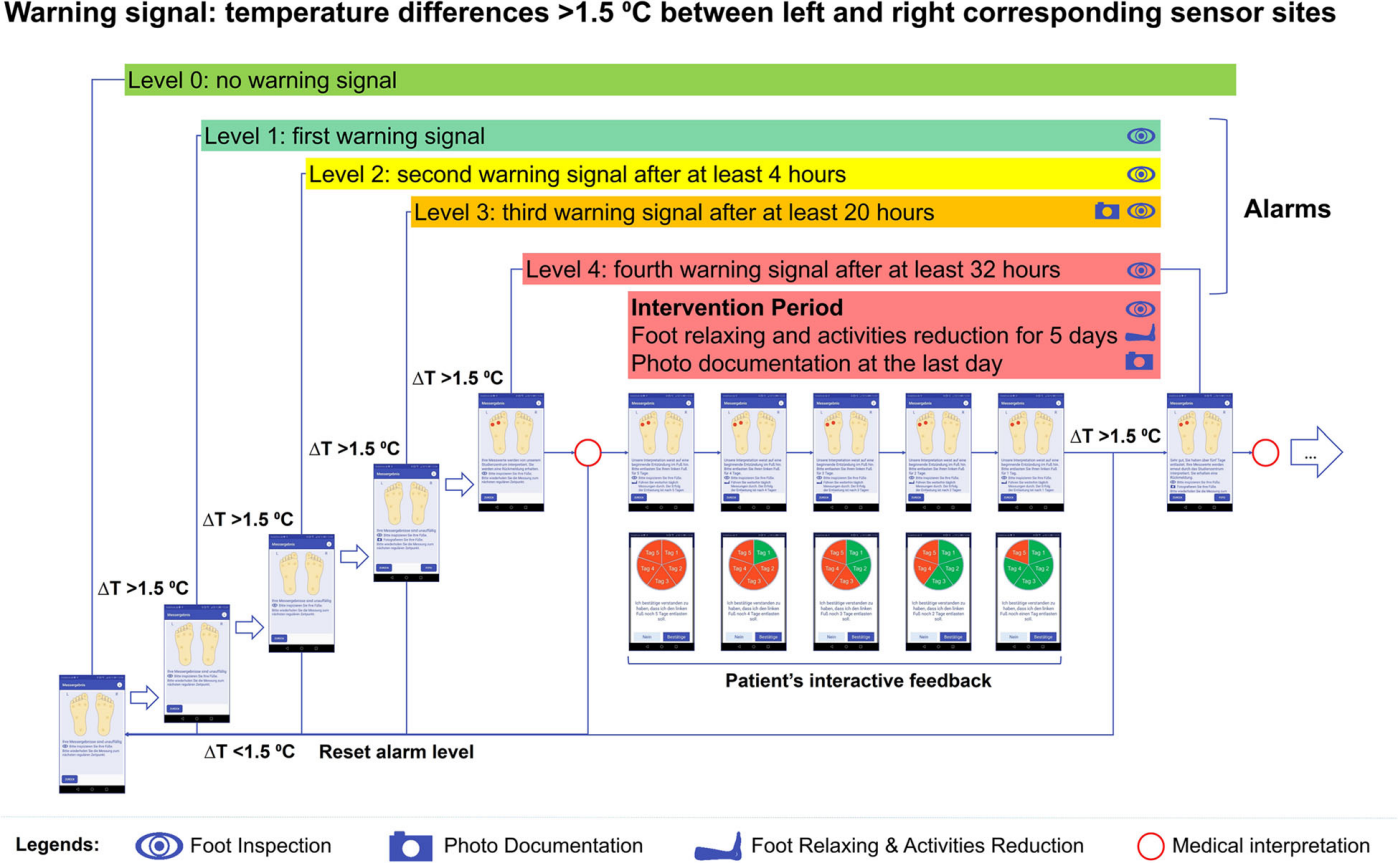
Alarm algorithm and related intervention measures. In the algorithm, a “warning signal” will be prompted if temperature differences are > 1.5 °C between left and right corresponding sensor sites. The following levels have been implemented: level 0 = no “warning signal”; alarm level 1 = first “warning signal”; alarm level 2 = second “warning signal” after at least 4 h; alarm level 3 = third “warning signal” after at least 20 h; and alarm level 4 = fourth “warning signal” after at least 32 h. Alarm level 4 encompasses that the study physician interprets the alarm. Other eventful alarm levels (“noticeable,” “confirmation,” or “intervention”) will be automatically reset, if the previous “warning signal” disappears. The related intervention measures include (1) reminding the patient of regular foot care and temperature measurements (every alarm level), (2) performing foot photo documentation (alarm level 3 and 4), (3) interpreting warning signals (alarm level 4), (4) prescribing an intervention period of five days foot relaxation and reduction in daily activities, and (5) recording patients’ interactive feedback during this period

For this study, an alarm algorithm with five alarm levels was developed that can be visualized on both the study server (for the physician) and the SPDFA (for the patient). In the algorithm, a “warning signal” will be prompted if temperature differences are > 1.5 °C between left and right corresponding sensor sites (Fig. 4). The following levels have been implemented: level 0 = no “warning signal”; alarm level 1 = first “warning signal”; alarm level 2 = second “warning signal” after at least 4 h; alarm level 3 = third “warning signal” after at least 20 h; alarm level 4 = fourth “warning signal” after at least 32 h. Only the study physician can reset alarm level 4 to level 0 after evaluation. Other eventful alarm levels (levels 1–3) will automatically reset to level 0 if the initially detected “warning signal” is no longer reinforced. The above-mentioned alarm level is not one sensor-specific alarm but reflects the highest alarm level of all six pairs of sensor sites. Based on these alarm levels, the intervention measures vary from physician to patient.

**Fig. 5:**
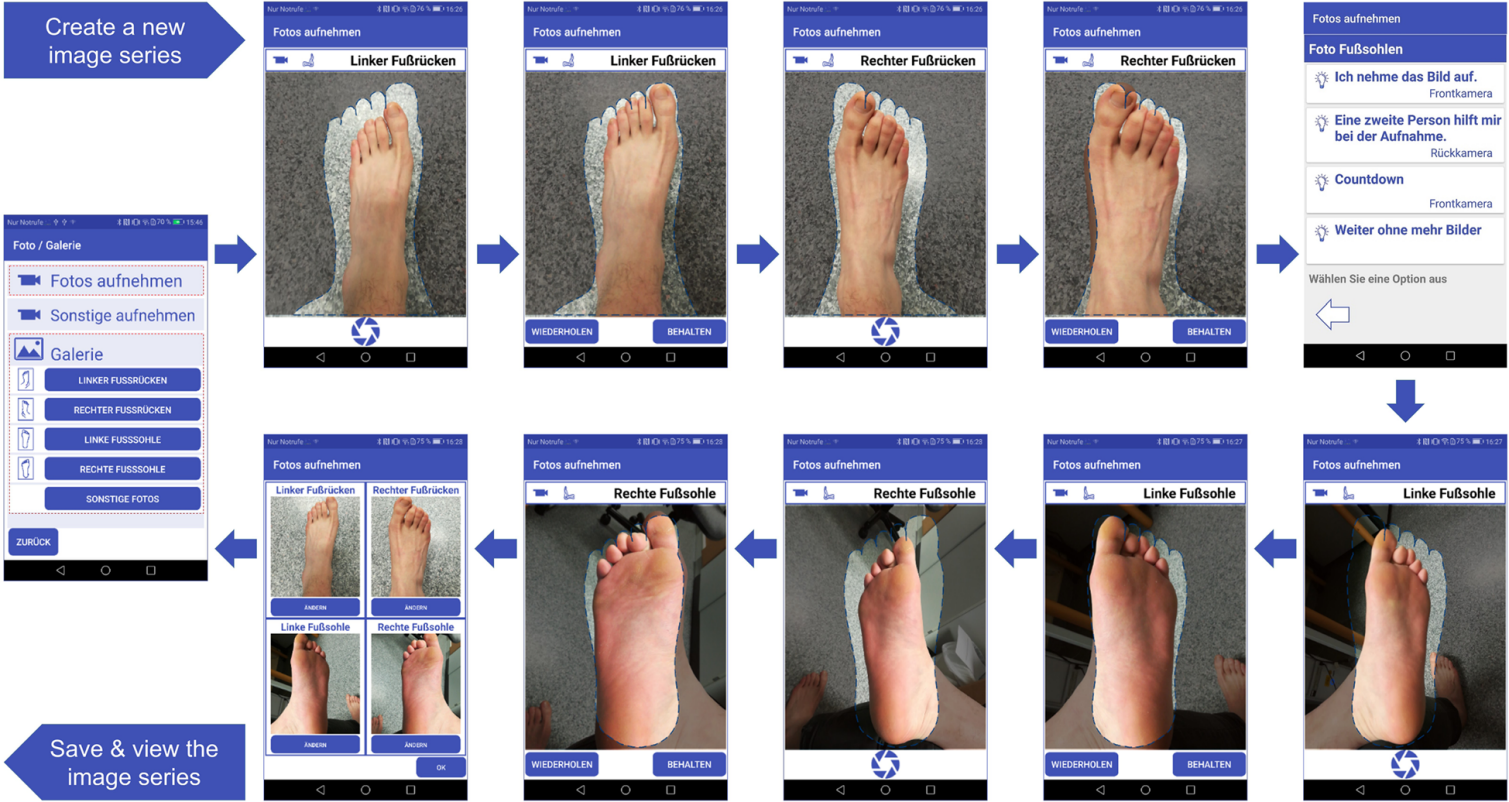
*Flow diagram* to take a photo series (four pictures) of each foot from the plantar and dorsal sides. The foot mask helps the patient to capture standardized foot images. Additional features such as a countdown function and a supporting selfie-stick are implemented to simplify the process especially in elderly patients with physical impairments

**Fig. 6:**
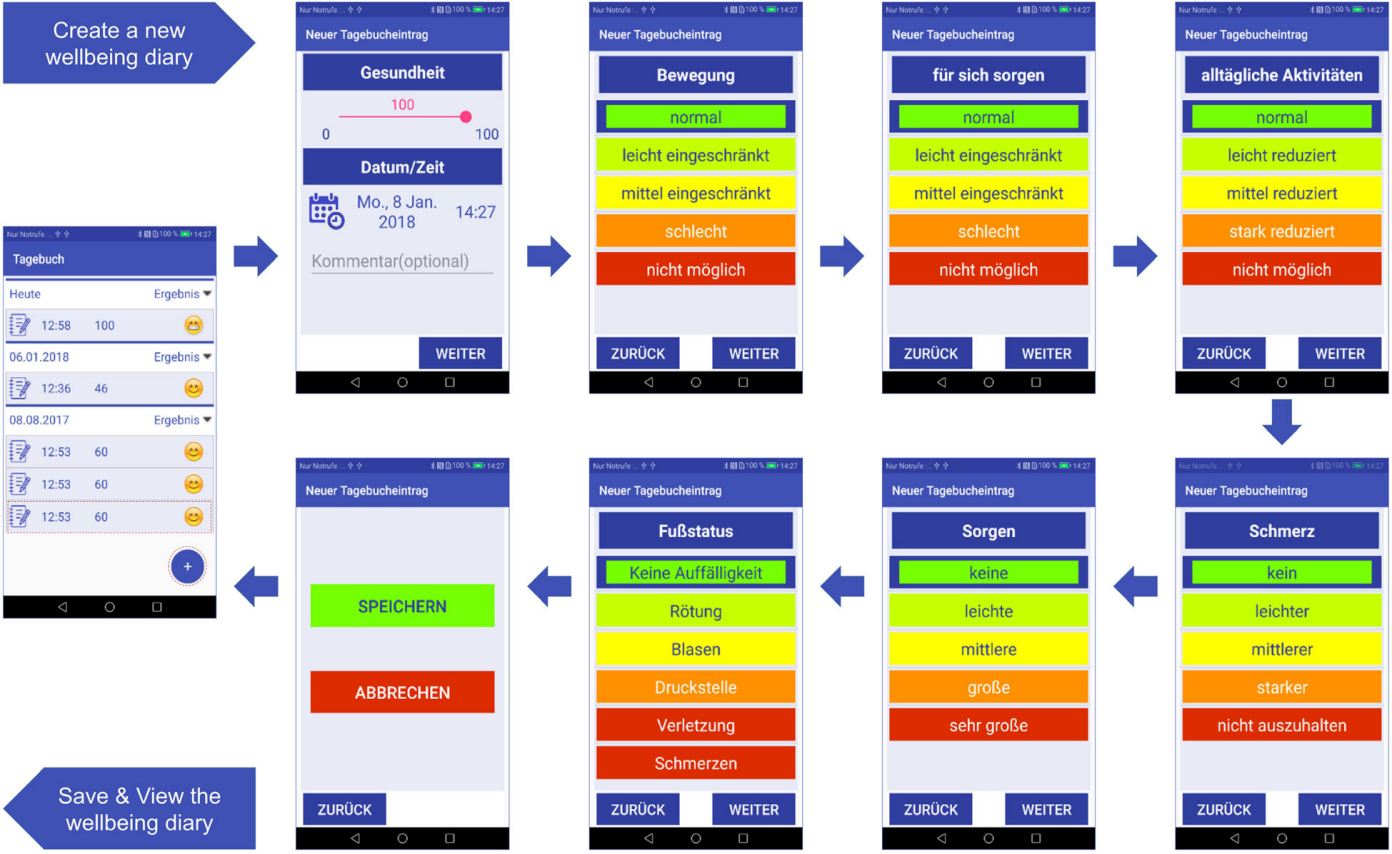
*Flow diagram* on health self-evaluation within the SPDFA. The interactive wellbeing score is based on the standard WHO-5 questionnaire30, which contains five items: mobility, self-care, usual activities, pain/discomfort, and anxiety/depression. Additionally, the assessment of foot status helps the patient to record the precursors of ulcer development, such as redness, blister formation, pressure sores, lacerations, pain, inflammation, and any other wounds

For the physician, on the study server side, the first notification for the physician will occur at alarm level 3. This means that the alarm has to be confirmed in repeated measurements for at least 24 h. At alarm level 4, the physician will interpret the temperature data together with the patient’s past temperature recordings, foot photos (Fig. 5), the interactive diary (Fig. 6), medical history, and laboratory data. If the alarm is confirmed to be a true positive ulcer alarm, the physician will prescribe an intervention period via server that requires the patient to relax his foot and to reduce daily activities for five days. In the case of an assumed “false positive alarm,” the physician will reset the alarm level 4 to level 0.

In contrast, on the SPDFA, the patient will be reminded to perform foot inspection and temperature measurements at every alarm level. At the respective alarm levels (1–3), the patient will receive a classification result as “uneventful.” Then, at alarm level 3, the patient will be asked to take a photo series (four images; of each foot from the plantar and dorsal sides; Fig. 5). At alarm level 4, the patient will be informed that his measurements will be interpreted remotely by the physician. If the physician recommends an intervention from the server, the patient will be continuously informed to relax his foot and to reduce daily activities for five days. The patient will also be requested to confirm that he follows the advice to relax his feet and reduce daily activities with an interactive dialog and a countdown sequence.

Following the five-day intervention, the physician will evaluate the collected data together with the patient’s feedback and the foot images taken on the last day during this period. Depending on this evaluation, the physician will determine whether another intervention period is required or if a doctor’s visit is needed.

### Control group

Patients randomized to the control group will be educated for optimal foot care by a study physician at the entry into the study and will be supported on any aspects of foot care during the study course.

At the study visits at 6, 12, 18, and 24 months, the same interviews and physical examinations as the intervention group will be performed to determine the foot status and possible ulcer formations.

### Adherence to the treatment plan

For patients in the intervention groups, the transmitted data of the intelligent insole is automatically stored in the study server. If no data are collected for seven days in a row or < 17% of all measurement points within a three-month observation period, the server generates a note for the study team. Thus, it can be clarified by telephone callback or in the context of the study plan why data were not collected.

### Intervention provider

The study coordinator and the study advisor are both physicians with > 2 years of professional experience as practicing physicians in internal medicine and diabetology. All other physicians involved in the study have professional expertise and experience in the conduct of clinical studies. The principal investigator and the study coordinator are responsible for staffing and training of the study team. All study-specific responsibilities are defined and authorized in the delegation log by the principal investigator. The training activities are documented in a training log.

### Outcome measures

#### Primary outcome measure

The primary outcome is occurrence of foot ulceration at any point during the 24-month study after visit 0. The severity level of foot ulcerations is classified according to the Wagner-Armstrong classification [30]. Any lesion will be considered as an ulcer in the sense of the primary endpoint (≥ Wagner level 1). Primary endpoints are also assessed according to time to onset of event and to the total number of events (ulceration) in the groups.

#### Secondary outcome measures

The following have been defined as secondary outcomes:

1. Adherence to the daily two-time temperature measurement based on data acquisition with the app
2. Report on alert frequency in the intervention group based on data acquisition with the app
3. Detection of slow temperature drops as an indicator of blood supply disorders (at daily intervals temperature changes are recorded and evaluated by the study physician: when temperature in the forefoot or whole foot drops considerably compared to the contralateral sensor data (> 1.5 °C) and reach ambient temperature levels an additional visit to the study center will be initiated to test for changes of blood supply) safety-relevant instructions concerning diabetes, the equipment (insole) or others that are evaluated by the study protocol: frequency of AEs and SAEs
4. Precursors of the primary endpoint: redness, infections, or wounds in the foot area (the precursors are recorded by AEs/SAEs reports, follow-up and unscheduled visits, as well as patient’s report through photo documentation of the SPDFA)
5. Quality of life according to the WHO-5 score [31, 32] at visits 1, 2, 3, and 4

### Sample size

Based on previously reported studies, we assumed a 20% ulcer occurrence rate over two years to be a conservative estimate for the control arm (where in case of a higher occurrence rate, the sample size becomes smaller) [15, 33, 34]. For the estimation of the treatment effect, we assumed a hazard ratio of 2.8 in accord with the study of Armstrong et al. [15]. Sample size calculations by use of log-rank test were based on a type I error probability of 5% (two-sided) and a power of 80%, with a drop-out rate of 20% over a two-year follow-up period per patient. This resulted in a calculated required number of cases of 147. Therefore, we plan for an inclusion of 150 patients for the intervention arm (300 patients in total). Sample size calculation was performed using the software nQuery + nTerim 4.0 (Statistical Solutions Ltd., 2015).

### Statistical analysis

#### Primary endpoint

The primary endpoint “time to onset of the first ulcer” will be analyzed using Cox regression for the intention-to-treat population. Regressors are the treatment arm, age (in years), gender, risk class, and degree of neuropathy. The decisive test is the test adjusted to the other influencing variables for the influence of the therapy arm (a = 0.05, two-sided). The adjusted hazard ratio of the treatment, including a 95% confidence interval, is calculated as the corresponding effect estimator. Secondary analyses pertain to the same analysis but in the per-protocol population.

In addition, in the intention-to-treat population, the ulceration rates for both treatment arms and the associated odds ratio are determined using the Mantel-Haenzel test with the risk class as stratification, whereby the patients are included in the analysis regardless of the actual follow-up period. In addition, the highest Wagner classifications of an ulceration observed for each patient (possibly 0 if no ulceration) are compared between the two therapy arms using the Mann–Whitney U-test, whereby these analyses are performed separately for the two risk classes.

#### Secondary endpoints

The precursors of ulceration are analyzed analogously to the primary endpoint.

The score values of the quality of life at the different time points are analyzed by means of mixed models for repeated measurements, whereby the four stratification factors from randomization (risk group, gender, age and the degree of neuropathy) are also included as influencing variables in addition to the therapy arm. The main comparison refers to the time of 24 months.

AEs and SAEs are recorded separately by treatment arm and risk class. In logistic regression models, a comparison between the therapy arms (insofar as the type of AEs/SAEs is not coupled to the experimental therapy arm) is made with the occurrence of at least one event per patient as the target and the same influencing variables as in the analysis of the primary endpoint.

The usage data of the insoles and the corresponding app are first extracted for the patients of the experimental arm from the automatic machine recordings and aggregated in the sense of the corresponding secondary endpoints (Prof. Dr. med. Siegfried Kropf, Institute for Biometry and Medical Informatics, Otto-von-Guericke-University Magdeburg).

All analyses are carried out using the software packages SAS or SPSS.

### Monitoring, quality control, and data management

Standard policies of the Otto-von-Guericke University Magdeburg for the development and review of the protocol will be followed, as well as policies related to adherence, safety procedures, and information management. The Trial Steering Committee will be composed of the study coordinator, co-investigators, principal investigators and the ethics committee of the Otto-von-Guericke University Magdeburg, who will provide trial oversight.

According to the harmonized ICH Guideline for the EU (ICH Theme E6) [35], “original data” is all information from original records and certified copies of the original records of clinical findings, observations or other activities in a study, and the necessity for the traceability and evaluation of the study. The principal investigator will provide access to original data (original records or certified copies) for all authorized persons listed in this protocol or included in the delegation log.

According to our Data Monitoring Plan, we will perform quality control at multiple stages, which include: (1) the use of manuals for data collection; (2) weekly meetings with study nurses; (3) updates concerning training about protocol procedures; (4) duplicate data entries to the database; and (5) the ongoing review of the descriptive statistics for the trial data by the principal investigators with quality control review of selected data, looking for inconsistencies, missing data, and outliers. The databases will be encrypted and password-protected to ensure confidentiality. Close cooperation between the study coordinator, the data manager, and other members of the study team will be established to allow the tracking of the progress of the study to solve problems that arise during implementation and to address other issues in time.

If the competent state authority or even the higher federal authority schedules an inspection, the same conditions apply as for an audit.

## Discussion

This study makes three principal contributions concerning the prevention of DFUs. First, the introduction of sensor-equipped insoles to promote daily home-based measurements of foot temperatures. Second, the implementation of a telemedicine structure with a smartphone app to measure foot temperatures, provide photo documentation, and evaluate wellbeing (quality of life) using an interactive diary. These collected data will be transferred to a remote server for interpretation and adjustment of intervention measures. Thus, our system appears much more sophisticated and provides more reliable data compared to simple thermometric approaches. Ultimately, intelligent predictive models for DFUs will be built with the collected sensor data and interpretations, which may support medical care providers.

Instead of using a thermometer (TempTouch; Xilas Medical, San Antonio, TX, USA) [14, 15] or thermal imaging devices (TempStatTM) [16], our study innovatively introduces the sensor-equipped insole to help diabetic patients to perform daily home-based monitoring of foot temperatures. The insole can easily be inserted into house slippers or shoes and may record the temperature data continually for several hours if required. It provides a more convenient and comfortable way for frequent temperature measurements.

The telemedicine structure implemented in our study comprises a remote server as core controller in the study center and the smartphone application (SPDFA) as data collecting terminal. With the SPDFA, patients can immediately comprehend the initial analysis results of their measurements. Sensor data will be transmitted from the SPDFA to the study server, together with the initial evaluations, the requested photo series (at alarm levels 3 and 4) (Fig. 5), and a self-assessment about wellbeing and foot status using our interactive diary (Fig. 6). Compared to the approach by Lazo-Porras et al., patients in our study do not need to identify the pre-defined alarm signs by themselves and consult the study physicians or nurses for timely interpretation [16]. For medical interpretation, our approach provides more information by means of photo documentation and using a wellbeing score, instead of only collecting temperature data. The study server stores the data and provides physicians with an interface to visualize the status of the patients and to interpret the ulcer alarms. In the case of a confirmed alarm, the study server can exchange data with the SPDFA to perform suitable intervention measures for the patient and to collect the patient’s interactive feedback during intervention periods. This approach will evaluate the effectiveness of activity reduction in order to delay or even avoid the development of DFU. Based on this concept, efficiency and timely interventions will be significantly improved. In addition, our alarm algorithms with stepwise graded alarm levels are able to test and verify various temperature warning measures (apart from only measuring temperature differences between left and right corresponding sensor sites) [16], time intervals between two alarm levels, individual corrections based on baseline data [17], or even different asymmetry thresholds [25].

Based on these collected data and clinical interpretations, intelligent predictive models might be built in the future for machine learning algorithms. With the development of such algorithms, intelligent telemedicine technologies have already proven to be one of the most cost-effective solutions for the early detection of DFU. As exemplified in the study of Goyal et al., deep learning methods for real-time DFU localization were applied to an extensive database of 1775 images of DFUs. The deep learning model showed great potential in the real-time localization of DFUs on an NVIDIA Jetson TX2 and a smartphone app [36]. The data collected in the present study will be important to test for an alarming system with a preset temperature threshold, compliance of diabetes patients to a bi-daily recording rhythm, and the challenges of picture recordings with a mobile app. Therefore, a whole package of innovation is brought to the intervention group participants; however, an entire telemedicine system with auto-response of the database recording system is not yet intended. The study physician interprets the data at 24-h intervals.

In subsequent studies, we will be able to test for different thresholds concerning temperature and alarm evaluation. This will allow us to furthermore adjust algorithms to detect other temperatures abnormalities caused, e.g. by Charcot foot, or vascular insufficiency. Ultimately, machine-learning algorithms and decision tree classification will be used to train an automated predictive model of DFUs with the data that are collected in past periods.

We believe that the complexity of the retrieved data from our protocol offers the potential to tackle a difficult problem from a unique aspect and, therefore, possibly will have a substantial impact on DFUs prevention not only in Germany but also in many other parts of the world.

## Trial status

This manuscript is based on version 1.6 of the trial protocol, dated 18 February 2019. Recruitment for this study began on 30 January 2018 and should be completed by 30 December 2019.

At the time of submission, our study has already recruited 196 patients; 87 patients were randomized into the intervention group. Of the 87 patients, 72 are active by daily measurements of foot temperatures with our system. The study is widely known in the area of Saxony-Anhalt; currently, a growing number of people with diabetes are eager to participate in the trial.

## Additional files

Additional file 1: Patient information and declaration of consent. (PDF 464 kb)

Additional file 2: Training materials of best foot care for control and intervention arms. (PDF 471 kb)

Additional file 3: Instruction of the equipment for the intervention group. (PDF 3170 kb)

Additional file 4: SPIRIT 2013 Checklist: Recommended items to address in a clinical trial protocol and related documents. (DOC 125 kb)

## Data Availability

Not applicable.

## Abbreviations

ABI: Ankle Brachial Index
AEs: Adverse events
DFS: Diabetic foot syndrome
DFUs: Diabetic foot ulcerations
IWGDF: International Working Group on the Diabetic Feet
NYHA: New York Heart Association
SAEs: Serious adverse events
SPDFA: Smart Prevent Diabetic Feet Application
WHO-5 Score: World Health Organization Five Well-Being Index
