# Supplementary material for: Study protocol for a randomized controlled trial to test for preventive effects of diabetic foot ulceration by telemedicine that includes sensor-equipped insoles combined with photo documentation
Source: Trials. 2019 Aug 22;20:521. doi: 10.1186/s13063-019-3623-x (PMC6704693; doi:10.1186/s13063-019-3623-x)
Supplement: Supplementary file 1 — Patient information and declaration of consent. (PDF 464 kb) [file 13063_2019_3623_MOESM1_ESM.pdf]

Patient information and declaration of consent

UNIVERSITY CLINICUM MAGDEBURG A.ö.R.

Center for Internal Medicine

University Clinic for Nephrology and Hypertension,
Diabetology and Endocrinology

Director: University Professor Dr. med. Peter R. Mertens

Information sheet for participation in the study: *"Intelligent" sensor-equipped insoles for patients with diabetic neuropathy for the prevention of foot ulcers*".

Ladies and Gentlemen,

We invite you to participate in the above mentioned clinical study. First, we will present you with an overview of the project, before the planned investigations and the procedure are explained in detail. Our study physicians will also talk to you directly about the study. Please ask your study doctor if you do not understand anything or if you have further questions.

What's the point?

We would like to ask you for your consent to participate in a clinical trial. The study will be conducted by Prof. Dr. med. Peter R. Mertens, Director of the University Clinic for Nephrology and Hypertension, Diabetes and Endocrinology at the Otto-von-Guericke University Hospital in Magdeburg and in cooperation with Thorsis Technologies GmbH and mediXmind GmbH. The study will investigate over a period of 2 years whether a shoe inlay equipped with sensors can prevent foot injuries in diabetics with severe nerve damage.

Nerves are essential for the perception of our environment. Especially nerves in the feet have the task to give us information about touch, pressure and temperatures. If they are damaged, you may fall, receive (unnoticed) injuries, develop permanently open wounds and joint malpositions due to incorrect loading will follow. With the development of an intelligent insole, we are pursuing the goal of replacing lost information. Sensors take over the task of the damaged nerves. We will investigate whether the occurrence of foot ulcers can be avoided by feedback of pressure and temperature changes. A safe positive effect on ulcer formation cannot be guaranteed.

What is the difference between the intelligent insole and standard insole?

This clinical trial will investigate a new method of care that deviates from standard therapy and is not yet approved for your disease. Routine care for patients with nerve damage includes protective shoes with **ready-made or custom-made insoles that** orthopaedic shoemakers customize according to the physician's instructions. The

sole is worked out according to the shape of the foot and consists of materials that distribute the pressure forces as evenly as possible. However, punctual loads cannot be completely compensated in this way. Pressure loads that lead to the dreaded circulatory disorders and ulcerations are not always prevented. This is why the intelligent insole was developed. In contrast to conventional insoles, it is equipped with several wafer-thin pressure and temperature sensors as well as a data memory chip. The sensors are integrated into the sole in such a way that they do not exert any pressure and are not felt. You regularly send data on the state of health of your feet to a mobile phone. From there you can call up the current measurement results at any time. If, for example, the health of your feet is threatened by an incipient ulcer, your telephone will send a message to the study Centre. This will then contact you promptly.

How does the study work?

Initial examination:

First, we need to determine whether your health allows you to participate in this study. This requires an initial medical examination as well as questions regarding the history of your illness. To participate in the study, a diabetic nerve disorder should be present. A clinical examination serves to exclude circulatory disorders (missing foot pulse), foot deformities and currently open wounds. An advanced cardiac insufficiency or a heart attack occurring in the last three months are also exclusion criteria for participation in this study. You should also have sufficient vision and reading skills to comprehend the messages from your phone. If the initial examination shows that you meet the criteria, you will be included in the study. All necessary materials (mobile phone, sensor-equipped shoe insoles, slippers) can be obtained from the study Centre. All study participants are randomly divided into two groups. The first group will wear the intelligent insole twice a day (morning and evening) for 5 minutes each over a period of 2 years. The second group retains their previous care.

Instruction and equipment:

After the initial examination, the patient is briefed and equipped in a second meeting. All participants will be instructed, independently of the group, in the medically recommended daily examination of their feet and in the selection of suitable footwear. Patients in the first group will also receive a pair of slippers with an integrated intelligent insole and a smartphone. They are trained in the use of the sole and the smartphone and receive information on how to behave in the event of a hazard alarm.

Follow-up examinations:

Participants of both groups receive regular medical check-ups every 6 months at the University Clinic for Nephrology and Hypertension, Diabetology and Endocrinology in Magdeburg (4 visits in total). They include a medical interview, a foot examination including photo documentation. Shoe insoles are checked for functionality and replaced if necessary. At the end of the study, the intelligent insoles and smartphones will be taken back.

Are there risks, complaints and side effects?

According to human judgement the application of the intelligent insole is safe, the formation of sharp edges is excluded during their production by the shoemaker, an additional examination is not necessary.

What should be done if symptoms, side effects and/or injuries occur?
If any symptoms, side effects or injuries occur during clinical testing, you must report them to your study physician and stop testing.

When will the clinical trial be prematurely terminated?
You can revoke your willingness to participate and withdraw from the clinical trial at any time without stating a reason, and without this resulting in any disadvantages for your further medical care.

Your study physician will immediately inform you of any new findings that may become known in relation to this clinical trial and that may be of significance to you.

However, it is also possible that your study physician (or, if applicable, the sponsor of this clinical trial) may decide to terminate your participation in the clinical trial prematurely without first obtaining your consent. The reasons for this could be:

- They cannot meet the requirements of the clinical trial;
- Your study physician has the impression that further participation in the clinical study is not in your interest;
- The client makes the decision to discontinue the entire clinical trial or merely to terminate your participation prematurely, as far as this is ethically justifiable.

Data protection:
In addition to your personal medical record, the results of the examination are also recorded on a so-called study sheet, which - instead of your name - only contains a code number. The study form is kept electronically in the form of a database. In order to increase data security, your contact data is stored and evaluated separately from your medical data. MediXmind GmbH is responsible for data processing. The data encrypted in this form is passed on exclusively for statistical purposes, i.e. to evaluate the data. Access to your data is restricted to both the client and the study staff. These persons are bound to secrecy. The data is protected from unauthorized access. Due to legal regulations, certain persons (authorized third parties) have the right to inspect your personal data / medical records. This includes representatives of the client. Inspection / disclosure only takes place within the framework of the legally regulated tasks of the inspecting party, namely for the purpose of checking the data. These persons are bound to secrecy. The data in this clinical trial will be archived for 10 years. You can request information about your stored data at any time. You have the right to have erroneous data corrected. You have the right at any time to revoke your consent to the processing of your personal data or to have your data deleted, unless otherwise required by law.

Are there any costs for the participants? Is there any cost reimbursement or remuneration?
All study participants receive an expense allowance of 5 euros per visit for participating in the study. The study materials (smart phones, intelligent insoles) are made available to the intervention group free of charge.

Insurances
All patients are covered by the statutory insurance coverage for clinical trials to compensate for financial loss as a result of study-related damage to health. During

the duration of the clinical trial, any other medical treatment, except in emergency situations, should be agreed upon with the investigator. If there is any suspicion of health damage due to your participation in the study, the study physician must be informed immediately so that he can inform the insurance company on your behalf. You can expect to receive a copy of the notification. You can also inform the insurance company yourself.
The insurance was taken out with the HDI Gerling Group with the insurance number 26022013205. The insurance conditions were handed over. Special reference is made to §§ 6 (Services) and 14 (Obligations).

Where can you get further information?
If you have any further questions in connection with this clinical test, your study physician and his staff will be happy to assist you. Questions concerning your rights as a patient and participant in this clinical trial will also be answered.

Contact:
Phone Study Center: 0391-6721745

Study director:
Prof. Dr. med. Peter R. Mertens
Director of the Clinic for Kidney and Hypertension Diseases,
Diabetology and Endocrinology

UNIVERSITY CLINICUM MAGDEBURG A.ö.R.

Center for Internal Medicine

University Hospital for Kidney and High Pressure Diseases,

Diabetology and Endocrinology

Director: University Professor Dr. med. Peter R. Mertens

Declaration of consent

on participation in the study: *"Intelligent sensor-equipped shoe inlay for patients with diabetic neuropathy for prophylaxis of foot ulcers*".

I hereby confirm that I have been orally informed by the investigator, Mr. /Mrs. Dr. .... about the nature, significance, course, risks and implications of the intended clinical trial and have had sufficient time to consider my decision.

I have read the patient information, I feel sufficiently informed and have understood what it is about. My doctor has given me ample opportunity to ask questions that have all been answered adequately for me. I've had plenty of time to decide.

My consent to participate in this research project as a patient or test person is entirely voluntary. My family doctor / other treating physicians may be informed about my participation in this clinical trial in order to take this into account in my further treatment.

I have been informed that I can revoke my consent at any time without giving reasons, without this resulting in any disadvantages for my further medical treatment and medical care.

I have received a copy of the patient information and this signed consent form as well as a copy of the insurance conditions.

First name, surname **Patient**	Place, Date	Signature
(In block capitals)	(Personally)	

CHECK:

I informed the patient orally and in writing about the goal, duration and course of the clinical trial. I have given the patient written patient information and a copy of the consent form.

________________________	________________________	___________________
First name, surname **Physician** (In block capitals)	Place, date (Personally)	Signature

Privacy statement

As part of the above-mentioned clinical examination, medical findings such as hospital stays, meditation, medication, foot images and personal information (e.g. age, gender, weight and height) are collected, documented and stored. The collected data is first recorded in your study file. In addition, important data for clinical trials are recorded on electronic documentation forms and data carriers in pseudonymised form (i.e. encrypted by a code consisting of numbers and letters without stating your initials or year of birth) without naming your name.

In order to increase data security, your contact data is stored and evaluated separately from your medical data. mediXmind GmbH is responsible for data processing. The data encrypted in this form is passed on exclusively for statistical purposes, i.e. to evaluate the data.

The data is protected from unauthorized access. Due to legal regulations, certain persons (authorized third parties) have the right to inspect your personal data / medical records. This includes representatives of the client. Inspection / disclosure only takes place within the framework of the legally regulated tasks of the inspecting party, namely for the purpose of checking the data. These persons are bound to secrecy.

The data in this clinical trial will be archived for 15 years after the end of the clinical trial. You can request information about your stored data at any time. You have the right to have erroneous data corrected. You have the right at any time to revoke your consent to the processing of your personal data or to have your data deleted, unless otherwise required by law.

The data is used in accordance with the statutory provisions of the State of Saxony-Anhalt (Saxony-Anhalt State Data Protection Act). Participation in the above clinical trial requires your consent to the following.

You cannot be included in the study without your consent.

1. I agree that personal data, in particular information about my state of health in paper form, as well as on electronic data carriers are documented in the context of the clinical examination. If necessary, these may be passed on

pseudonymised (encrypted) to the head of the clinical trial and to persons authorized by him for the purpose of scientific data collection and evaluation as well as monitoring of the proper conduct of the clinical trial by an independent commissioned body. In the event of unforeseen / undesirable events to the competent ethics committee and the competent supervisory authorities.

2. In order to ensure that the above mentioned clinical trial is conducted properly, I agree that authorized and confidential representatives of the sponsor and the responsible supervisory authorities may additionally inspect the original records in the study center. For this purpose, I release the investigator from the obligation of medical secrecy.
3. I agree that my data will be archived for at least 15 years after completion or termination of the examination. My personal data will then be deleted unless legal or statutory retention periods conflict with this.
4. I agree that my attending physician will be informed about my participation in the clinical trial (if not desired, please delete).
5. The study-related research results are to be used in anonymous form in scientific publications.

______________________	______________________	______________________
First name, surname **Patient**	Place, Date	Signature
(In block capitals)	(Personally)	
