# Supplementary material for: Study protocol for a randomized controlled trial to test for preventive effects of diabetic foot ulceration by telemedicine that includes sensor-equipped insoles combined with photo documentation
Source: Trials. 2019 Aug 22;20:521. doi: 10.1186/s13063-019-3623-x (PMC6704693; doi:10.1186/s13063-019-3623-x)

Intelligent insole for diabetic foot ulcer prevention

Isabell.walter@med.ovgu.de
Ahmad.alhajjar@med.ovgu.de

Clinic for Nephrology and Hypertension, Diabetology and Endocrinology
Otto von Guericke University Magdeburg

Overview Lecture

What is **diabetic foot syndrome**?

How do **foot ulcers** develop?

Checklist for your **foot health**

Study: Smart Prevent Diabetic Feet

Our feet carry us through life

We spend about **25-57% of** our lives on our feet.

Totally, 150 million steps could circle the **earth for 3 times.**

An area of 10 x 10 cm^2 bears our entire body weight.

The foot must withstand approx. 450kg/100cm^2 pressure with each step

Bones of both feet make up almost a **quarter of the total bones of** the body

Dangers to our feet

Very dangerous:

Injuries (ulcers)

Significance for diabetics:
Foot injury at 2 - 10%.

Risk of foot injuries increases with nerve damage and previous ulceration (up to 60%/year)

Germany:
60,000 amputations per year
because of a diabetic foot ulcer

70% of all amputations are due to
of a diabetic foot ulcer

What's a diabetic foot?

Foot changes due to diabetes mellitus.
They **promote** the formation of **foot ulcers**.

Two causes:

vessel damage

Arteriosclerosis / Clots

Oxygen supply to the legs disturbed

nerve damage

Sensory disturbance (formication)

In the process:
loss of feeling
Disruption of muscle and skin nutrition with muscle cramps and muscle atrophy

nerve damage

Sensory disturbance (formication)

In the process:
loss of intuition
Disruption of muscle and skin nutrition
with muscle cramps and muscle atrophy

What happens when you can't feel your feet?

Two major problems with loss of intuition in the feet

1. injuries go unnoticed

2. incorrect loads lead to restricted circulation

You wouldn't notice the little stone in the shoe.

You feel no pain if you stand on one spot for too long.

ulcer formation due to mechanical stress

Causes:
Incorrect loading, loading always in the same place, injury

cornea formation

bleeding under the skin

ulceration
of the skin

Deep foot infection

How do foot ulcers develop?

1. skin changes

pay attention ...

How do foot ulcers develop?

1. skin changes

pay attention ...

dry skin

How do foot ulcers develop?

1. skin changes

pay attention ...

increased horny skin formation

How do foot ulcers develop?

1. skin changes

pay attention ...

SKIN CRACKS

How do foot ulcers develop?

1. skin changes

pay attention ...

NAIL FUNGUS

bruises

SIGN: REDNESS / SWELLING

bruises

SIGN: BLISTER FORMATION

cornea formation

callused cornea with
haemorrhage

Beginning of ulceration

Frequent locations of pressure sores/blisters/ulcers

especially the areas between the toes

Unnoticed violations

The lack of sensation of pain, for example, means that **small stones in the shoe can** no longer be detected in time.

One consequence is progressive, often inflammatory ulcers on the feet.

Ulceration

Checklist Foot Health

Prevention is better than cure!

Important measures

1. Foot inspection
2. Cream feet

} **on a daily basis**

3. Remove cornea
4. Nail care
5. Check shoes

Prevention

Take a **fixed time of day to** check and care for your feet, for example in the evening before going to bed.
A **hand mirror is** suitable for checking so that you can see all the areas of your foot well.

Checklist Foot Inspection

- Is your foot **swollen**?
- Is the skin **reddish**?
- Does the foot feel **overheated**?
- Do you see a **blister** or a **bruise**?
- Are there **calluses** or **corns**?
- Are there **injuries** such as cracks, scratches, stings or wounds?
- Is the skin in the **toe interdigits** yellowish and torn?
- Are the **nails** thickened and yellowish or whitish **discolored**?
- Is a **nail** ingrown?

Cream

Due to disturbed perspiration, diabetic feet are very dry and brittle. To maintain or increase the moisture and elasticity of the skin, you can use skin care creams or foams.

Especially suitable are **creams and foams that** contain fat are quickly absorbed and provide sufficient moisture. Many users prefer foams because they offer sufficient moisture, but do not burden the skin with too much fat, are less sticky and absorb more quickly.

Cream

- Care creams or care foams (foam creams)
- Omit toe interstices.

The foams usually contain three important ingredients:

1. **Urea**:	moisturizing<br>strengthens the immune function/ regenerative ability
2. **Pentavitin:**	natural ingredients of the cornea
3. **Panthenol**:	moisturizing<br>promotes wound healing

Skin protection

Good, good, good:

Prevent athlete's foot

In case of foot or nail fungus, you should contact your doctor.

Bad:

Corn plaster/tinctures

Remove cornea

Good
corneal sponge/pumice stone

Bad:
avoid sharp objects
(scissors, clippers, corneal raspses)

Nail care

(approx. every 2-4 weeks)

Good:

glass nail files

podiatrists

Bad

pointed objects (scissors, nail clippers)

Footbath

Prevention of:
Inflammations, fungi and cracks

Requires:
Thermometer,
Washcloth,
mild soap/wash lotion
bowl

Footbath

lukewarm water (**about 37°C**)

maximum three to five minutes
dry carefully afterwards

Beware of already existing wounds:
Avoid foot baths with iodine,
as it can damage the
healthy tissues in
wounds.

6 tips for buying shoes

Not too tight

Not too loose

ideally seam-free

firm soles

sufficient space for inlays

If possible buy in the evening

Insoles for diabetics

- Inserts ensure even pressure distribution,
- Check the degree of wear regularly,

Special diabetic shoes

Ready-made special shoes

Orthopaedic made-to-measure shoes (exist as slippers, street shoes, sports shoes and bathing shoes)

with :

Removable, ready-made soft cushion sole

Diabetes-adapted foot bedding

Last, but not least:
No matter what kind of shoes you wear - you should check every time before putting them on whether there are small objects like splinters or stones in the shoes.

Diabetic socks for the diabetic foot

Properties :

- antibacterial
- anti-infective
- anti-odour
- washable
- antifungal
- naturally

Additional functionalities of Special diabetic socks:

additional padding

compression stockings

- Compression stockings are a medical device that can be used to treat venous and lymphatic
- diseases, including the prevention of leg vein thrombosis.

- The thrombosis prophylaxis stocking (antithrombosis stocking) is used in hospitals and nursing homes to prevent thrombosis in bedridden and freshly operated patients.

- In patients with PADCs, prescription and use should only be carried out under medical supervision.

Taking wound healing seriously

Wounds you don't feel are not consciously disencumbered.
Stay in bed with wounds, **relieve** foot **as much as possible**
Alternative: forefoot or heel relief shoes

Study: *Smart Prevent Diabetic Feet*

Study procedure:

Follow-up rounds:

Training

1st round

2nd round

3rd round

4th round

Month: 0 6 12 18 24

Next regular appointment

When do I contact the Study Centre?

Preliminary stages of an ulcer (ulcer)
(skin redness, blisters, infections,
wounds in the foot area)
Ulcer (ulcer)

Inpatient admission (planned and unplanned)

Appointments

Contact:
0391/ 67 -21615
0391/ 67 -21745

Gladly at any time with further questions

Who's treating my feet?

Your general practitioner/diabetologist remains your **first point of contact**!

If there are any changes to the feet, **always contact** the **Study Centre**!

First aid possible for injuries in the study centre!

Clinic for Nephrology and Hypertension
Diabetology & Endocrinology, OvG University

Contact details

Clinic for Nephrology and Hypertension,
Diabetology and Endocrinology,
Otto-von-Guericke University Magdeburg

House 60 B, Level 2

Phone: 0391 /67-21615
0391 /67-21745

E-mail: Claudia.Piehler@med.ovgu.de
Isabell.Walter@med.ovgu.de
Ahmad.Alhajjar@med.ovgu.de

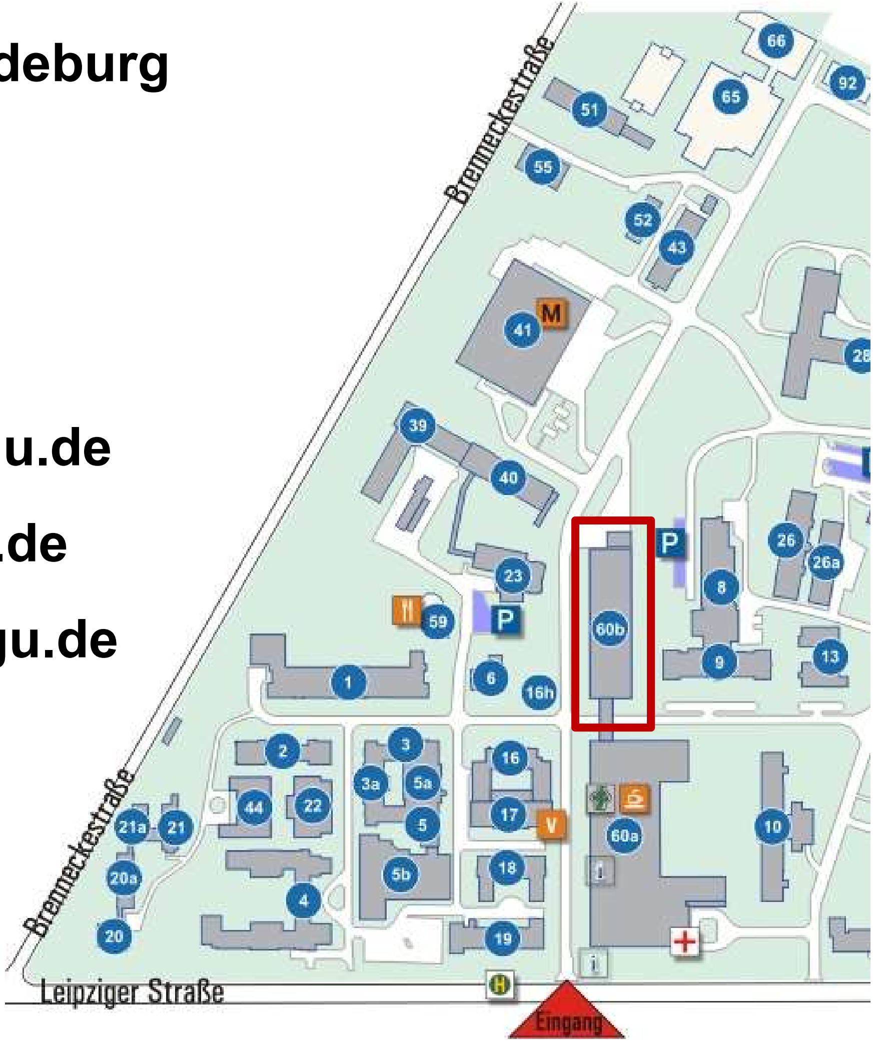

Supplement: Supplementary file 2 — Training materials of best foot care for control and intervention arms. (PDF 471 kb) [file 13063_2019_3623_MOESM2_ESM.pdf]
