# Supplementary material for: Study protocol for a randomized controlled trial to test for preventive effects of diabetic foot ulceration by telemedicine that includes sensor-equipped insoles combined with photo documentation
Source: Trials. 2019 Aug 22;20:521. doi: 10.1186/s13063-019-3623-x (PMC6704693; doi:10.1186/s13063-019-3623-x)
Supplement: Supplementary file 3 — Instruction of the equipment for the intervention group. (PDF 3170 kb) [file 13063_2019_3623_MOESM3_ESM.pdf]

Intelligent insole and Smartphone application

Isabell.walter@med.ovgu.de

Clinic for Nephrology and Hypertension, Diabetology and Endocrinology
Otto von Guericke University Magdeburg

In studies:

Daily temperature measurements can detect ulcers on the feet about five weeks before they develop.

Resting the feet prevents ulceration in 2 out of 3 cases.

We would like to support you,
Identify dangers to your feet in good time

10 minutes a day for your feet

How to measure correctly...

Morning

&

time interval: >4 hours

2x daily

Evening

Sitting

pay attention
same measurement conditions

Delivery of the study materials:

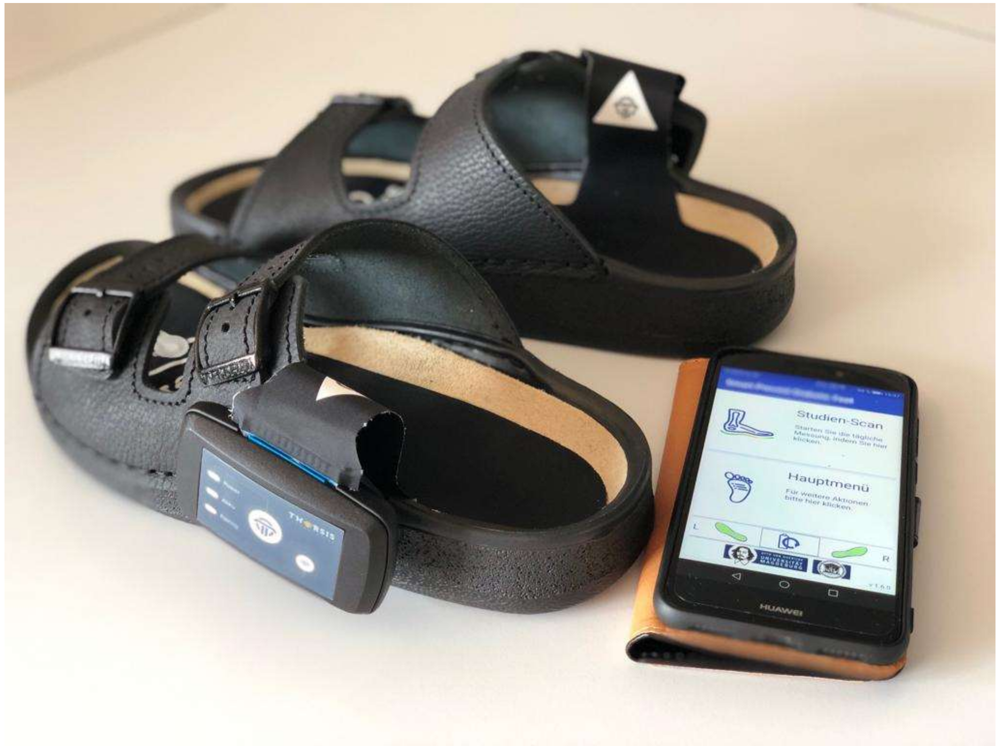

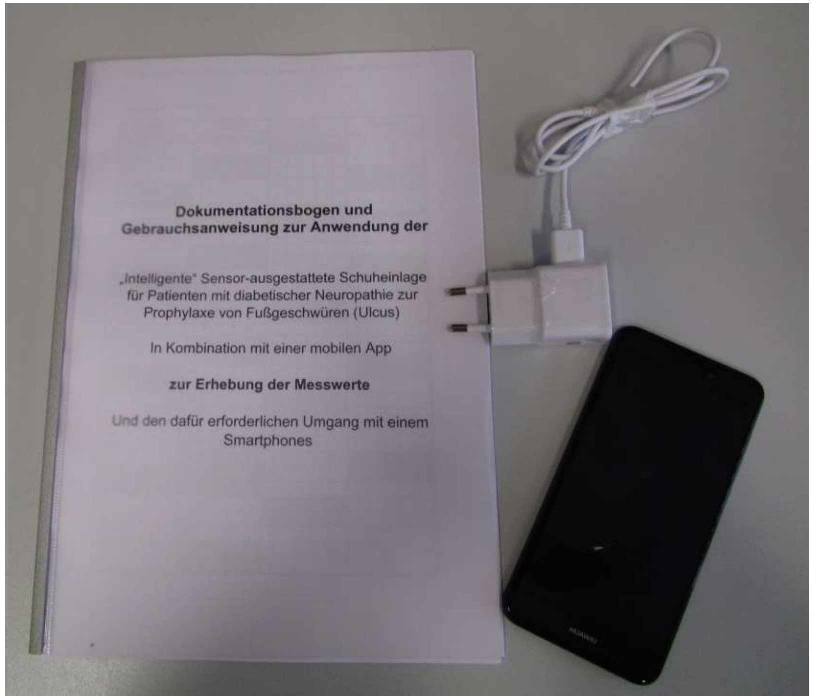

1 pair of slippers incl.
1 pair of intelligent insoles

Mobile phone incl. charger
Study handbook incl.
instruction manual

Please use equipment exclusively for study purposes!

Introduction of electronics

Buttons

To **switch on** the electronics, please press the “ON/OFF” button once.
A **0.5 sec green signal** of the “Power” LED indicates a successful start of the electronics.

To **switch off** the electronics, please press the “ON/OFF” button once again.
A **0.5 sec red signal** of the “Power” LED indicates a successful close of the electronics.

To pair the electronics to the smartphone via Bluetooth, please press the “Pairing” button once.
It will initialize a Bluetooth pairing mode (30 s). The “Pairing” LED will show orange during this period.

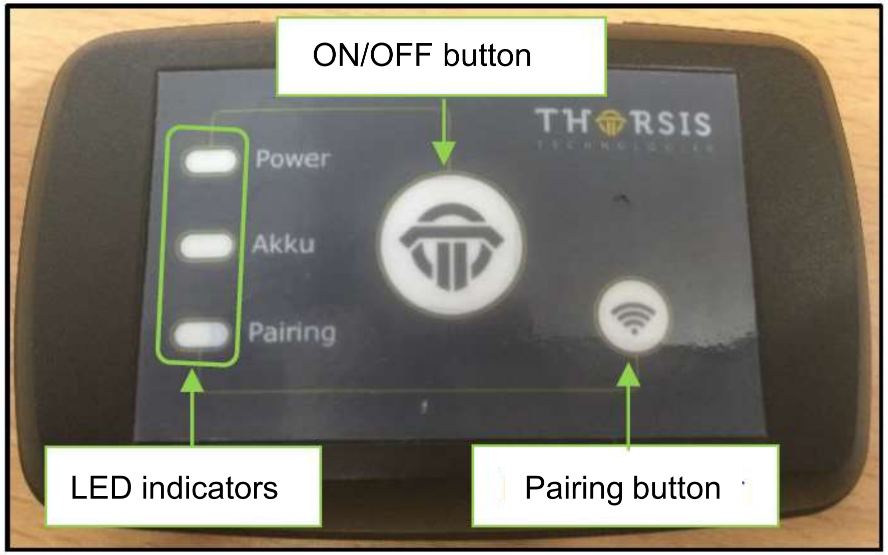

Introduction of electronics

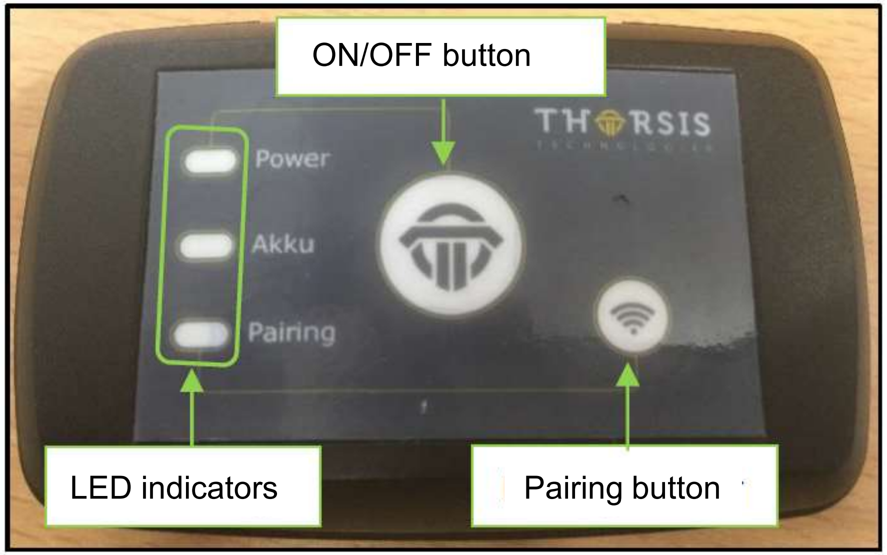

LED Indicators

Power (double LEDs)

- (green) start-up
- (red) shutdown

Battery (double LEDs)

- (green) The electronics are correctly connected to the charging cable. The appropriate charging voltage is applied.
- (red) The battery is charging. This LED goes out as soon as the battery is fully charged.

Pairing (simple LEDs)

- (orange) Insole is now ready for pairing. (duration approx. 30s)

Charging the electronic battery

Connect the two **electronic components to** the **socket using** the supplied **cables** and the **adapter**.

Pay attention to the **two-color** LED "Battery". It shows red and green (two colors) when the battery is charging. It **only** lights **green when** the battery is **full.**

LED display
(shows loading progress)

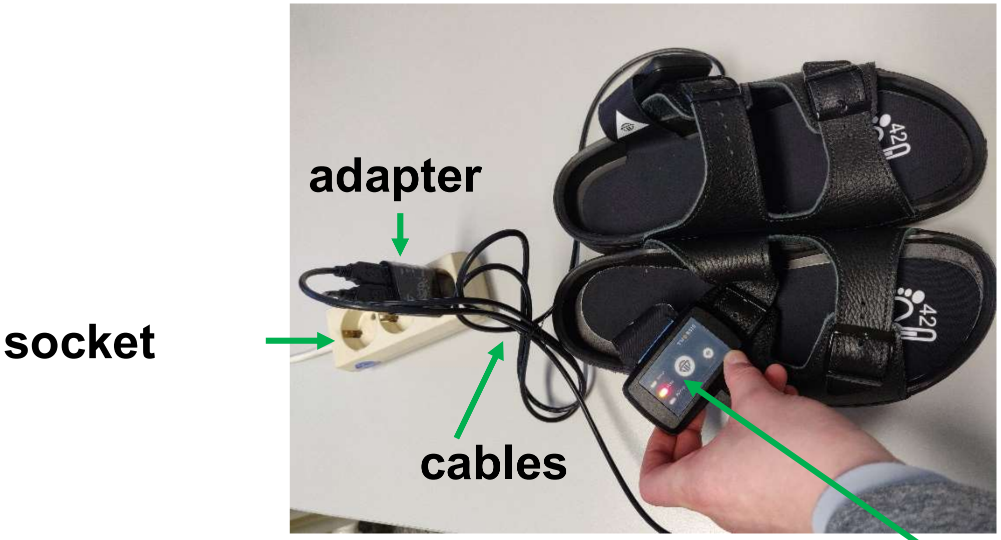

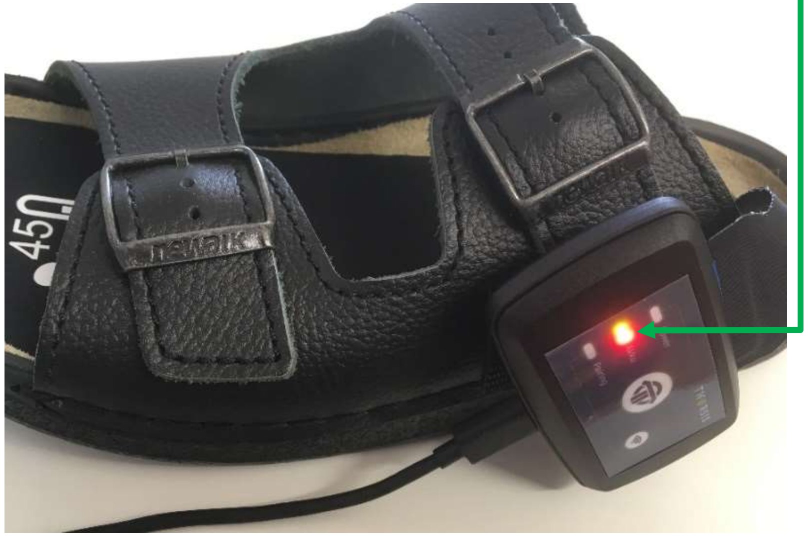

socket

electronic components

Application of the insole

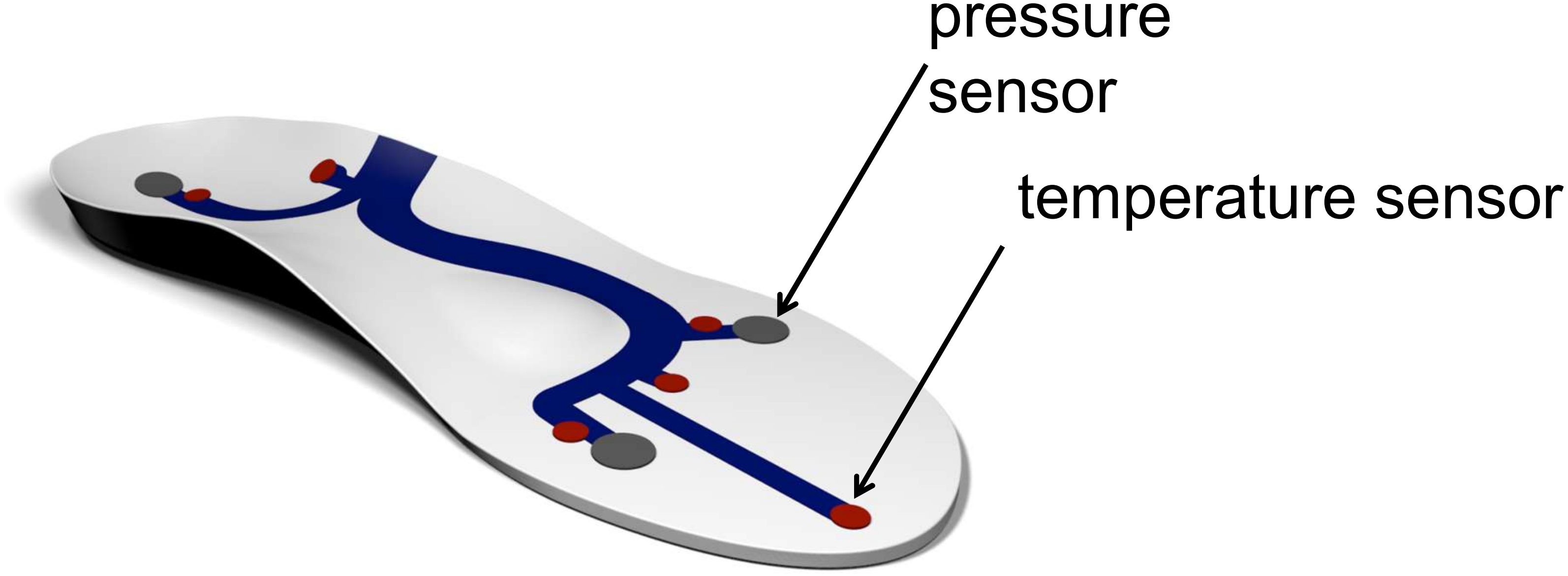

1. insoles

protect from moisture

2nd measurement

with socks

on intact skin

Study Smartphone

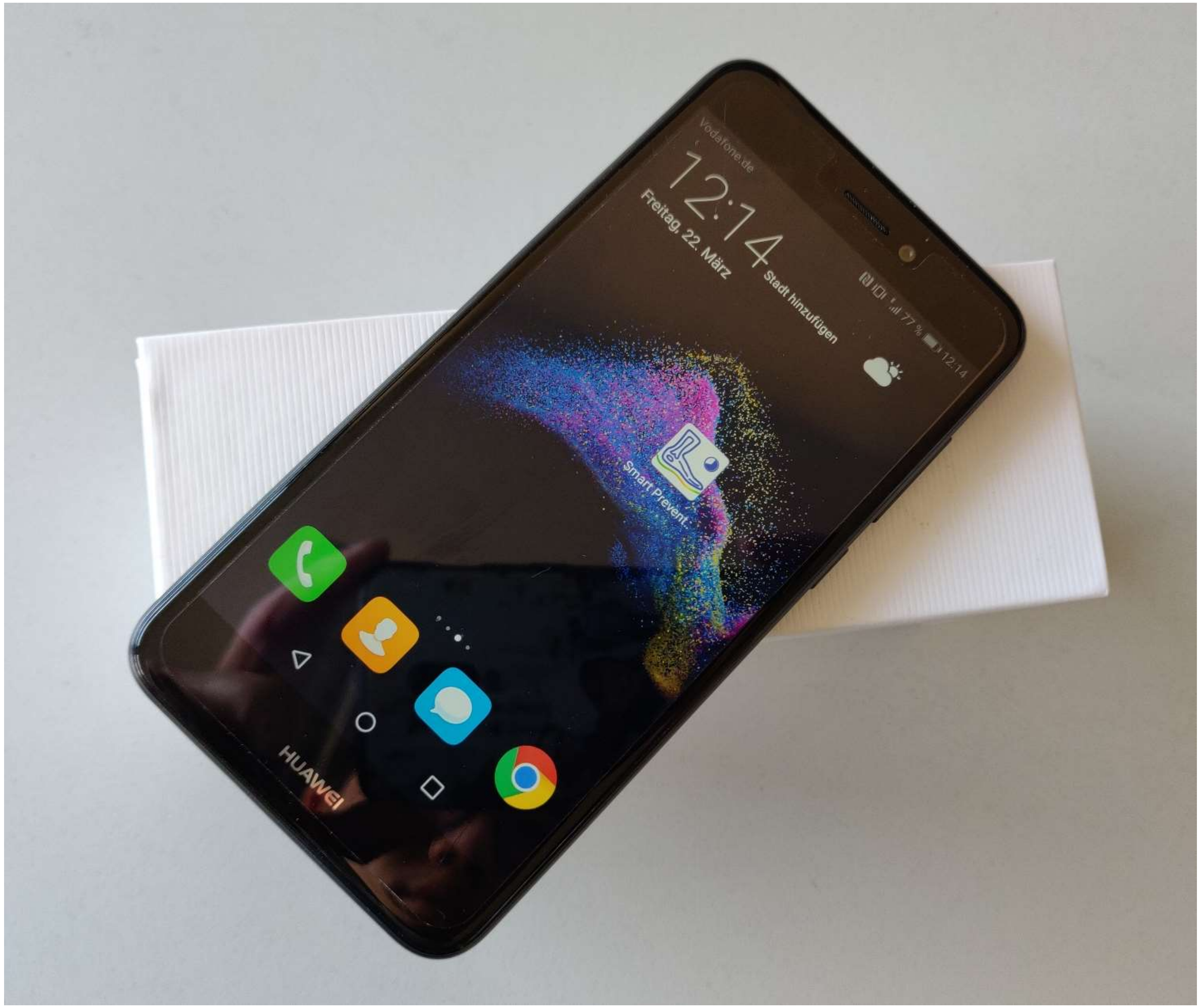

Study Smartphone: Push Button

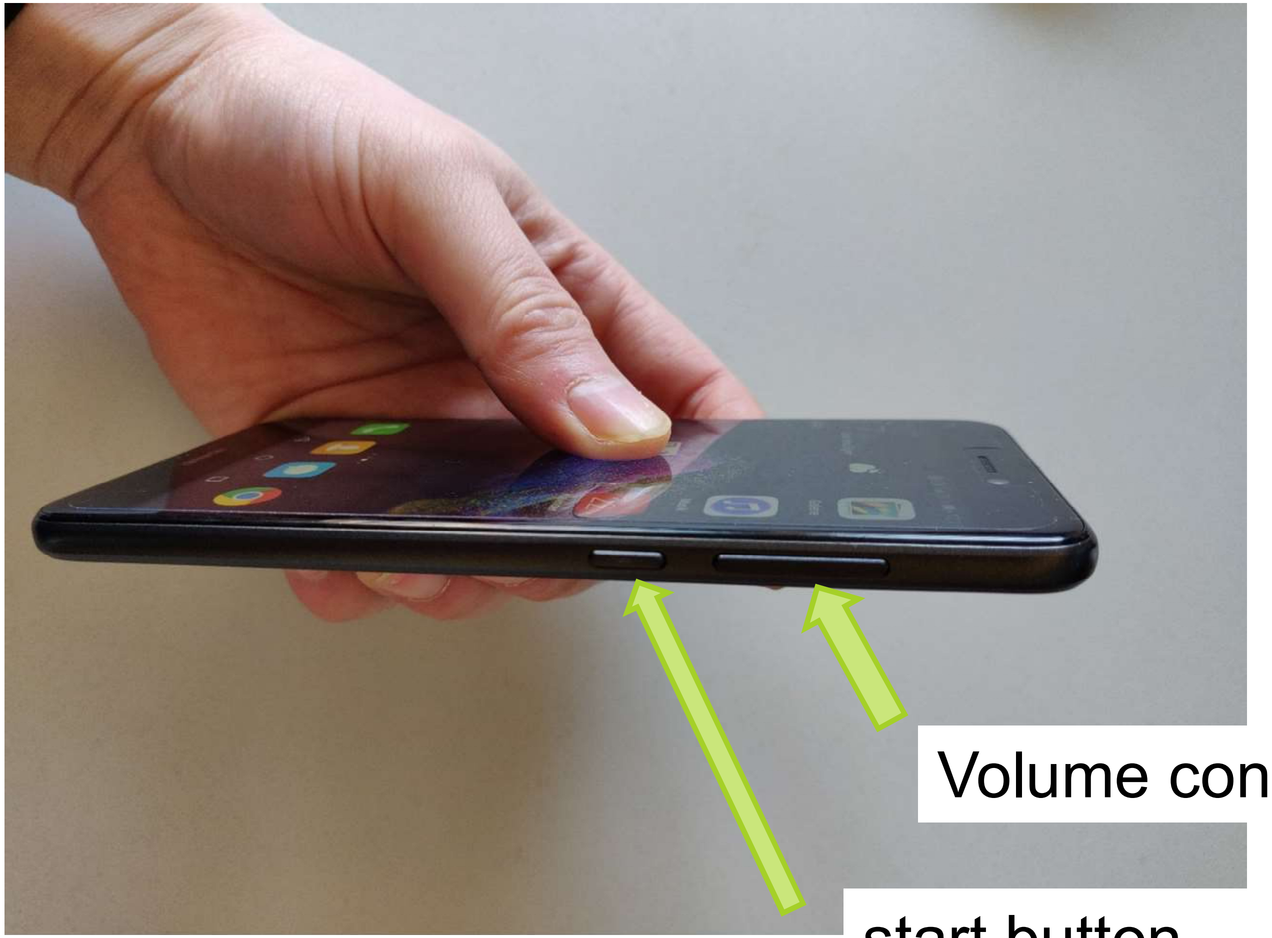

Smartphone: Charging the battery

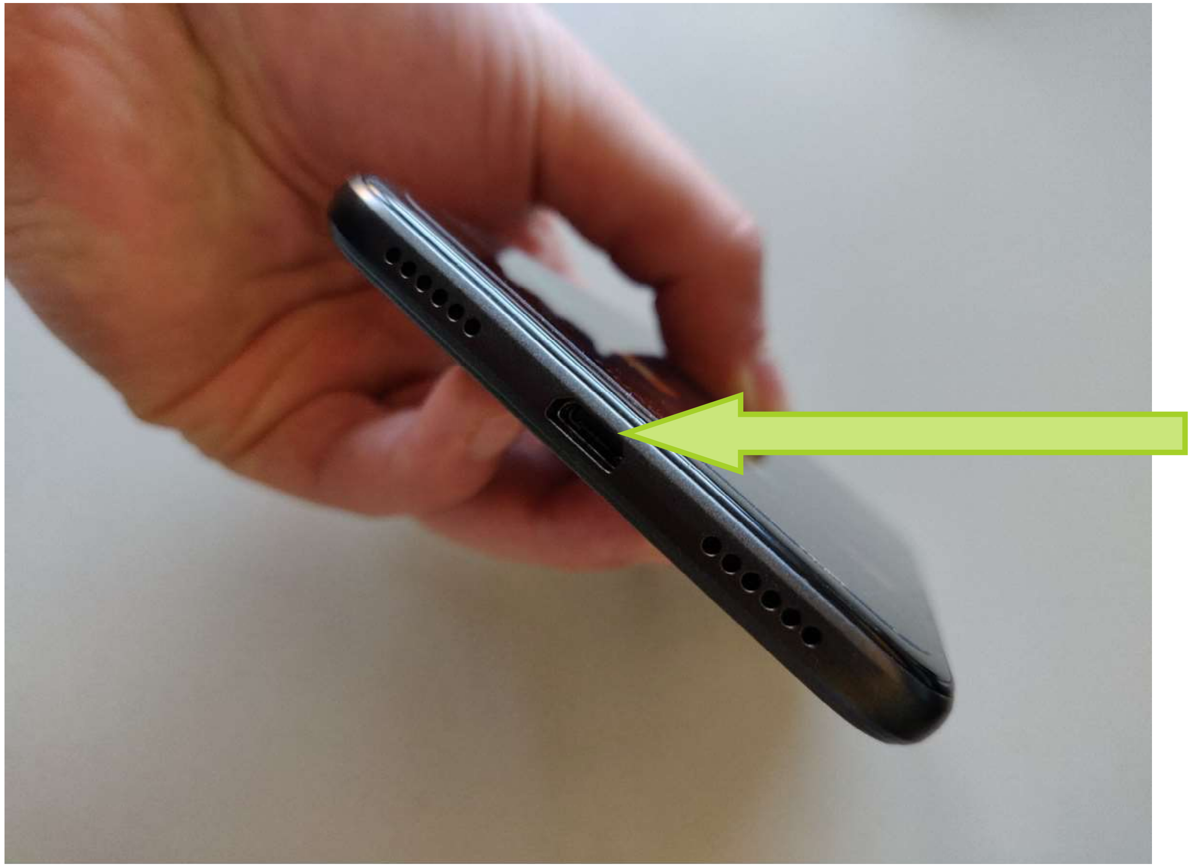

Connection for the charger

about: 1x daily
Duration: approx. 4h

Smartphone: Camera

front camera

rear camera

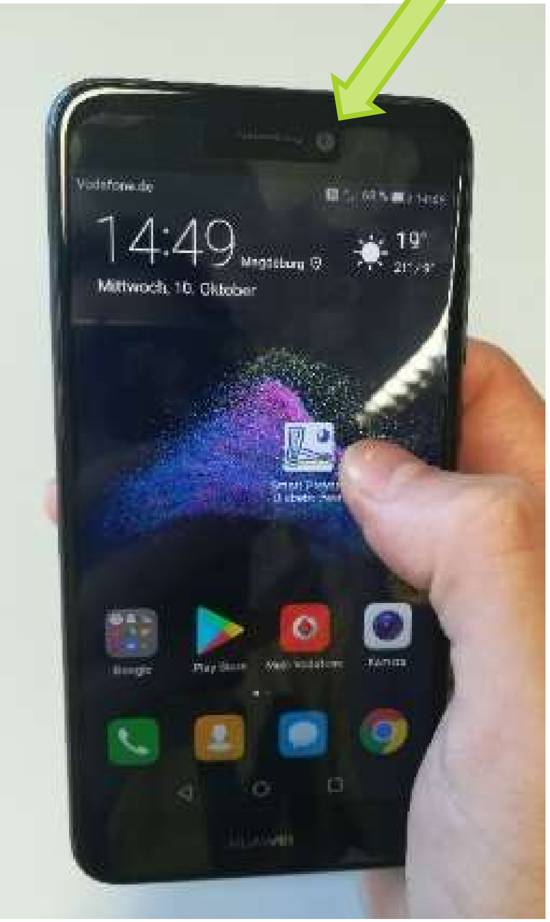

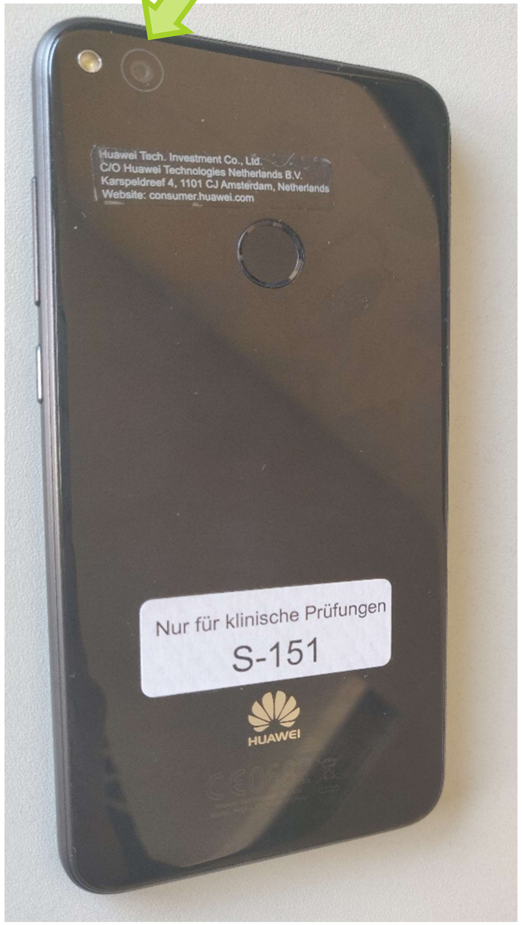

Smartphone: Menu

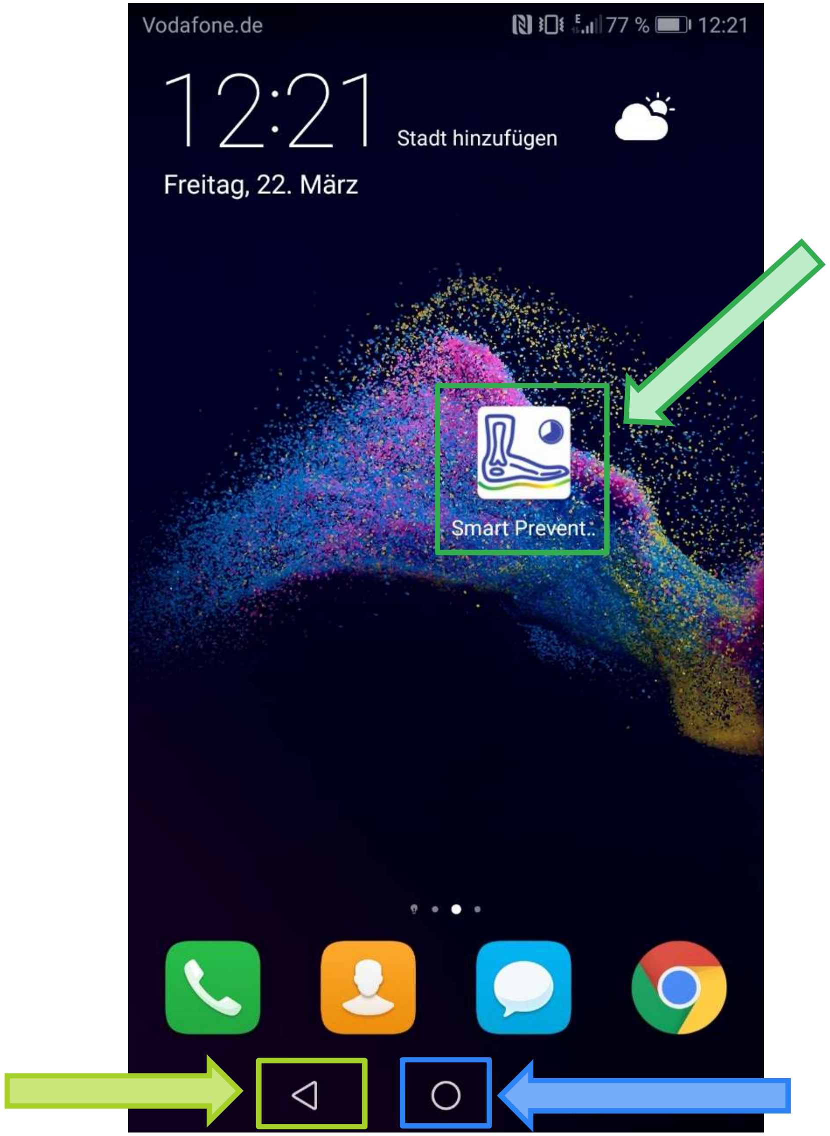

Study App

"Back button"

"Smartphone menu button"

Recommended order for carrying out an ulcer measurement

Step 1:
Start the smartphone.

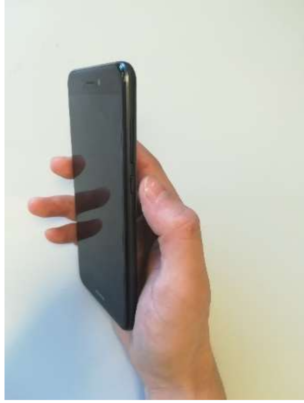
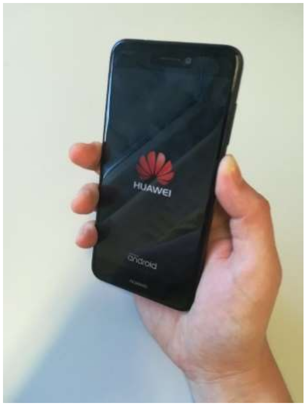

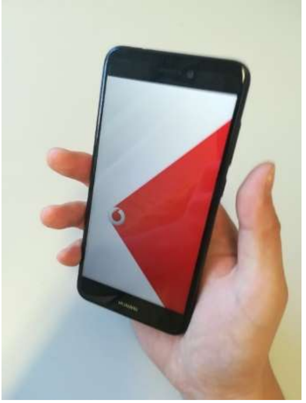

Switch on mobile phone...

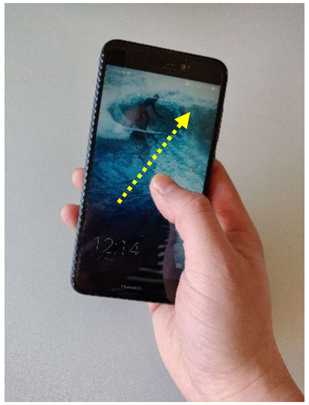

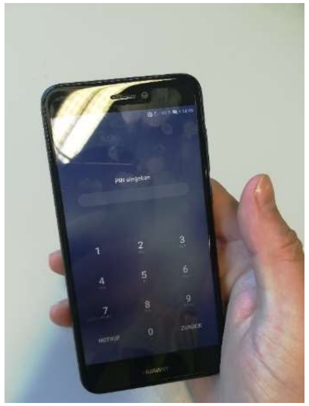
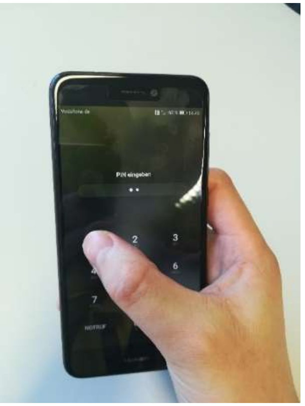

Wipe the screen with your finger

Enter PIN (1111)

Recommended order for carrying out an ulcer measurement

Step 2:
Launch the Smart Prevent Diabetic Feet application

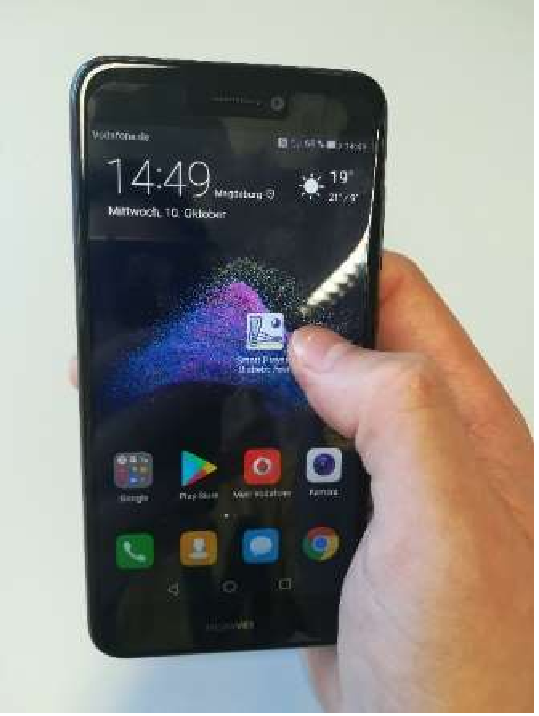

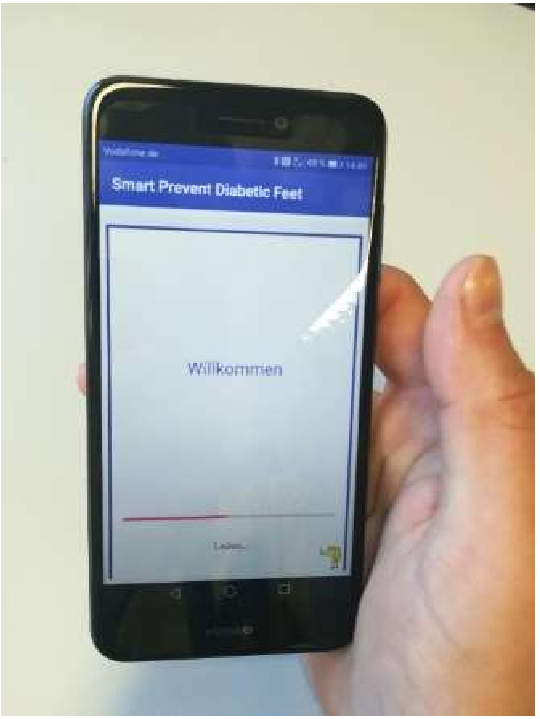

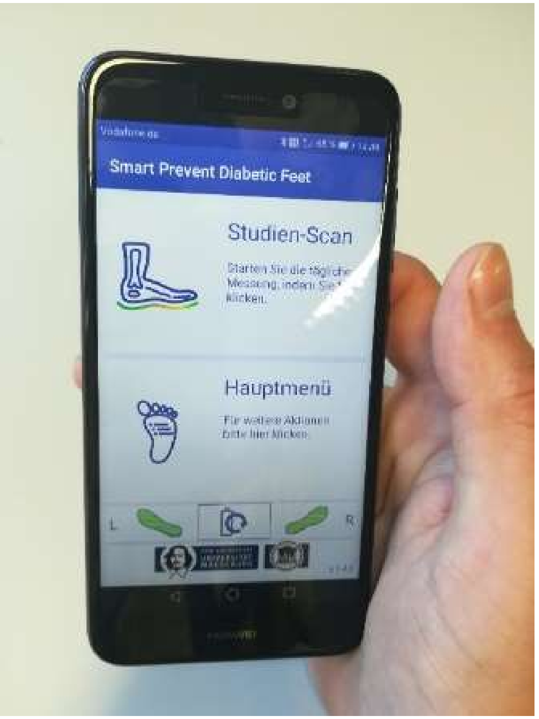

Open Smart Prevent Diabetic Feet App

Recommended order for carrying out an ulcer measurement

Step 3:
Switch on the electronics of the two insoles by pressing the central switch button (**ON/OFF button**).

The "Power" LED will flash **green**.

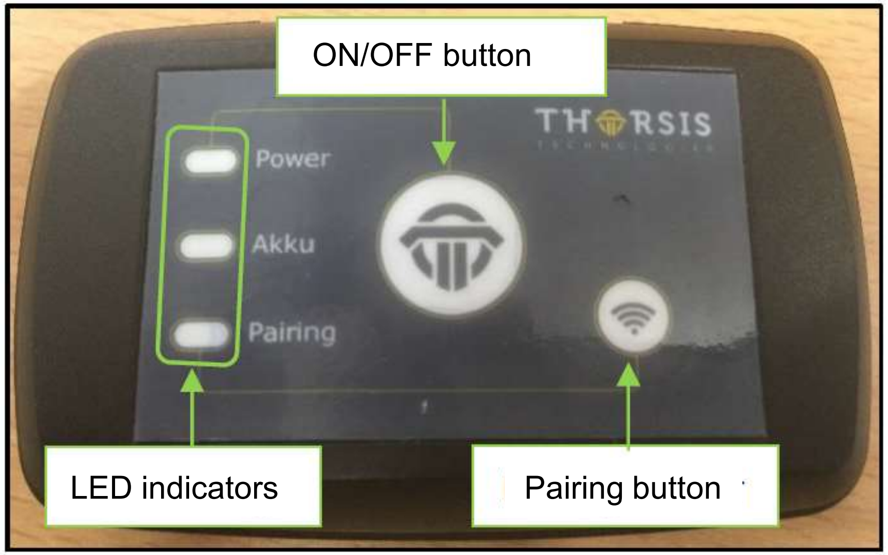

Recommended order for carrying out an ulcer measurement

Step 4:
Put on the study shoes with the inlaid intelligent insole.

Step 5:
Take a straight, relaxed sitting position with both feet on the floor.

Step 6:
Make sure that your feet cover all sensors of the two insoles. Check the position of your feet in the slippers.

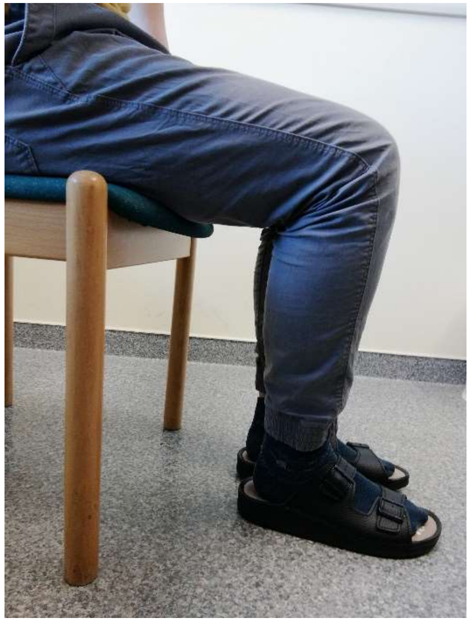

Recommended order for carrying out an ulcer measurement

Step 7:
Start the study scan (the temperature measurement) on the study app of the mobile phone.

Step 8:
The measuring process takes 3 minutes. For this period, please do not leave the sitting position and keep your feet calm in your shoes.

Step 9:
You receive your measurement result in the study app of the mobile phone. If necessary, follow the recommendations for action.

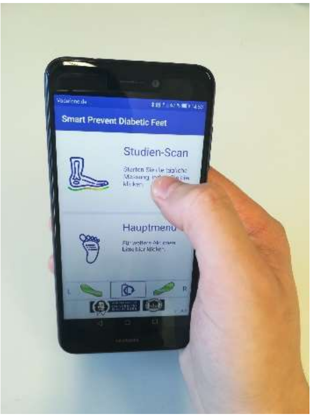

Start measurement

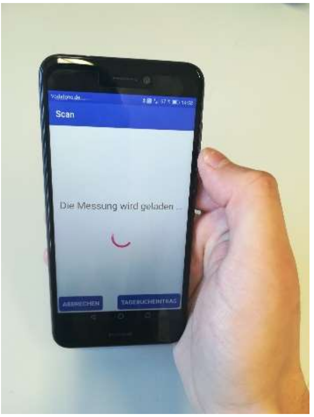

Measurement is loading

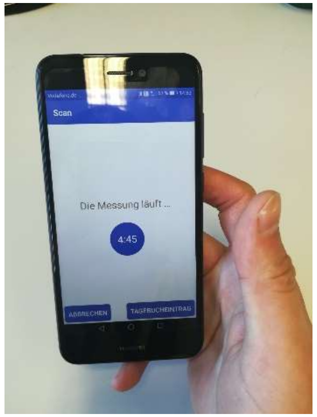

Measurement running 3 min

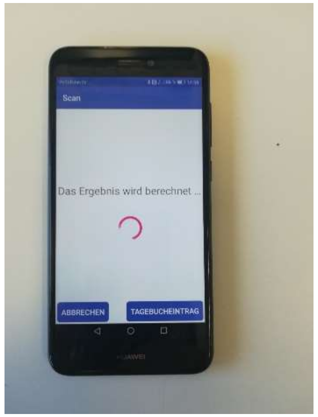

Result is calculated

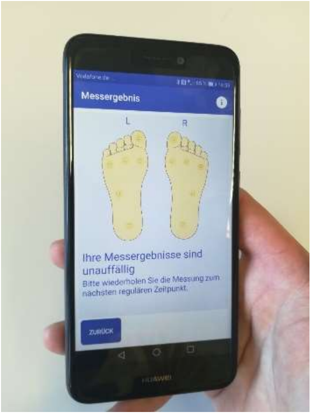

measurement result

Recommended order for carrying out an ulcer measurement

Step 10:
Switch off the electronics of the two insoles by pressing the central switch button again (**ON/OFF button**).

The "Power" LED will flash **red.**

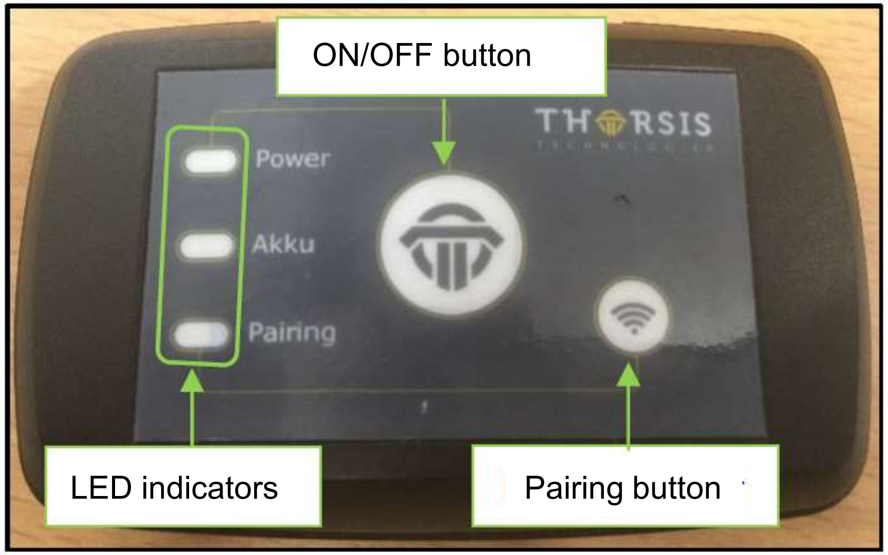

alarm gradations

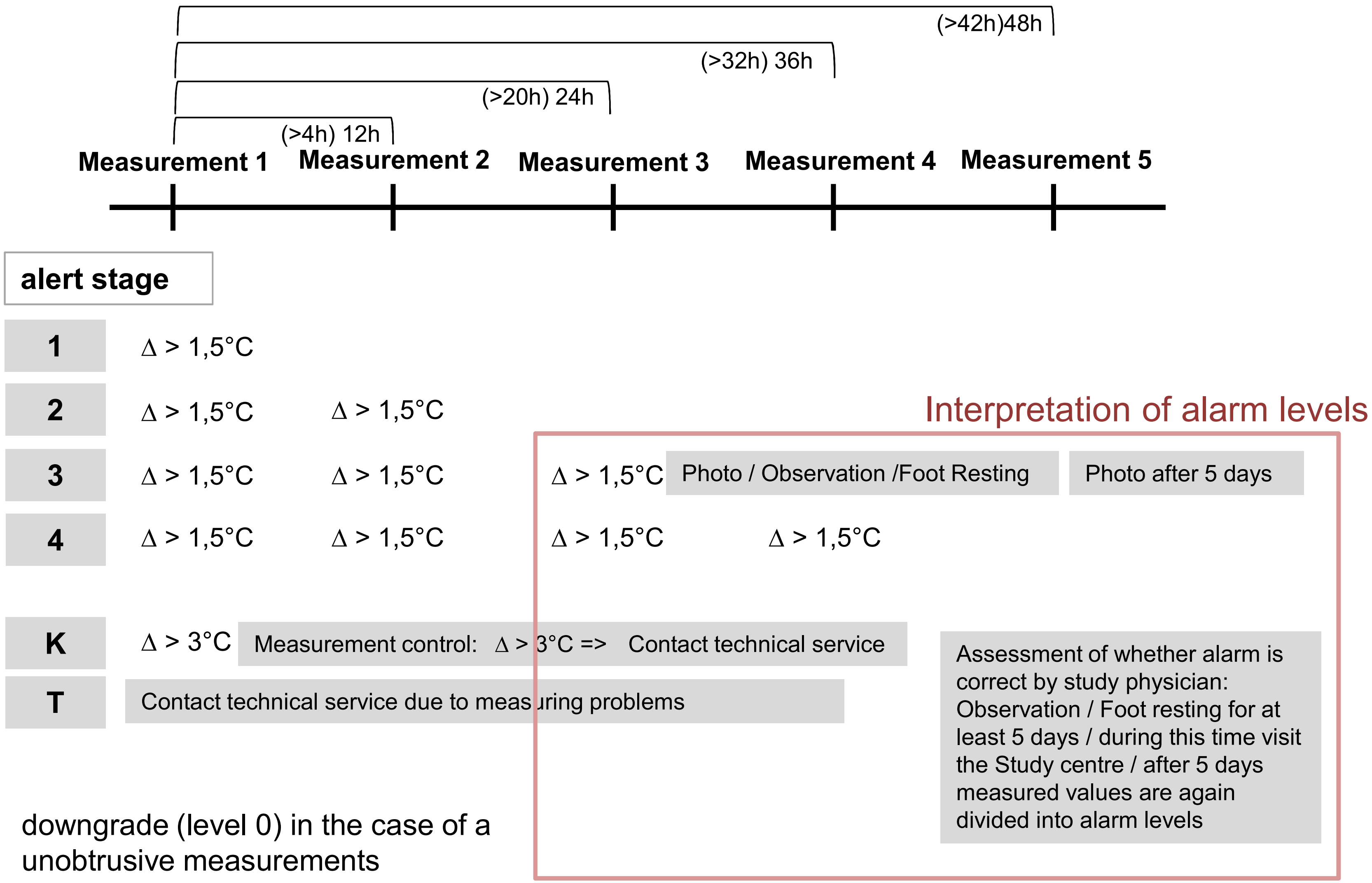

downgrade (level 0) in the case of a unobtrusive measurements
or after contact with a study centre
& all-clear there

additional app functions

mobile phone application

History of the

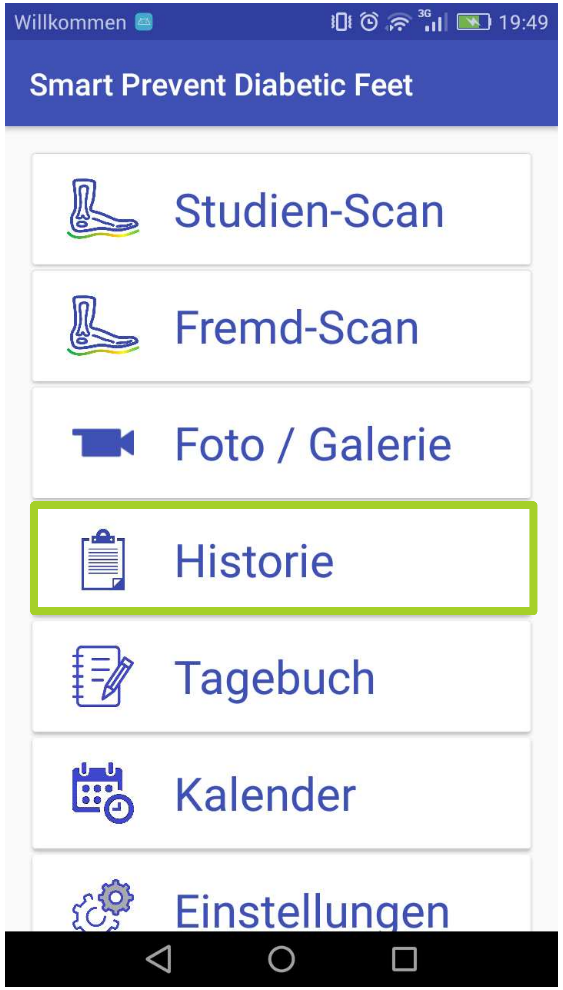

Viewing **temperature curves on** the feet

History of the

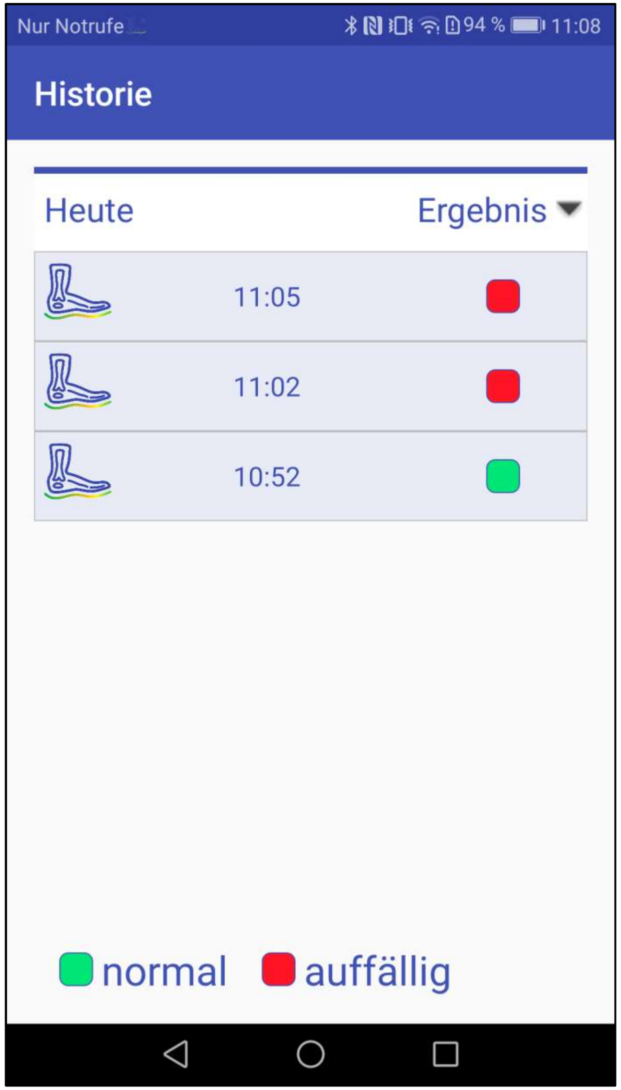

to view the measurement result

smartphone application

Result messages

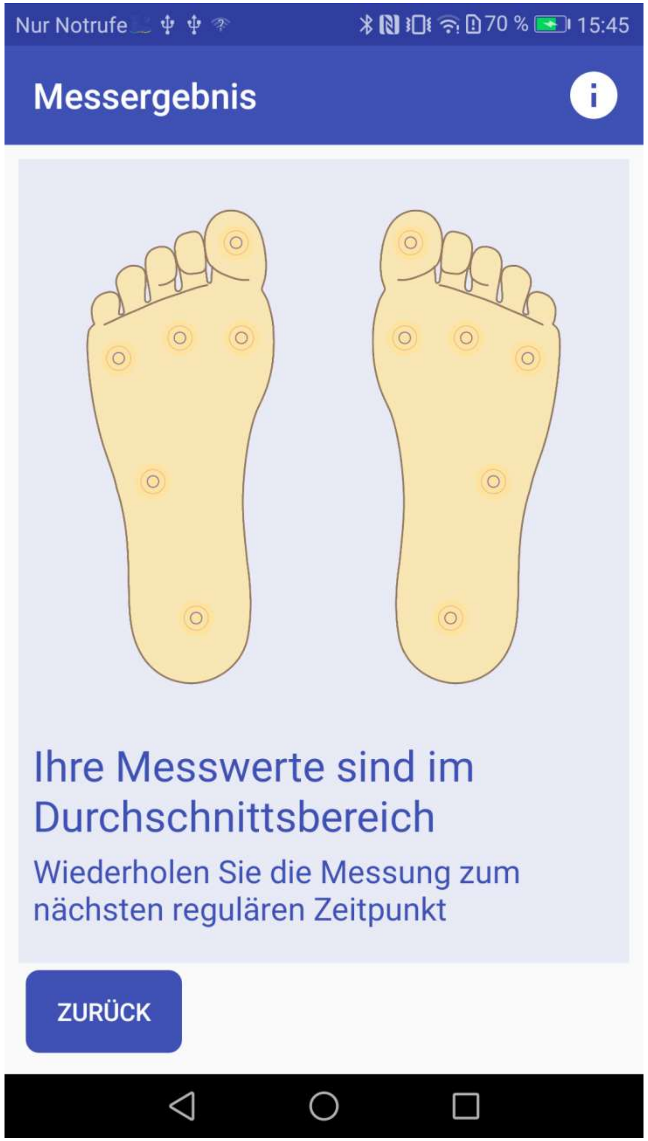

No abnormalities

(continue with next regular measurement)

History

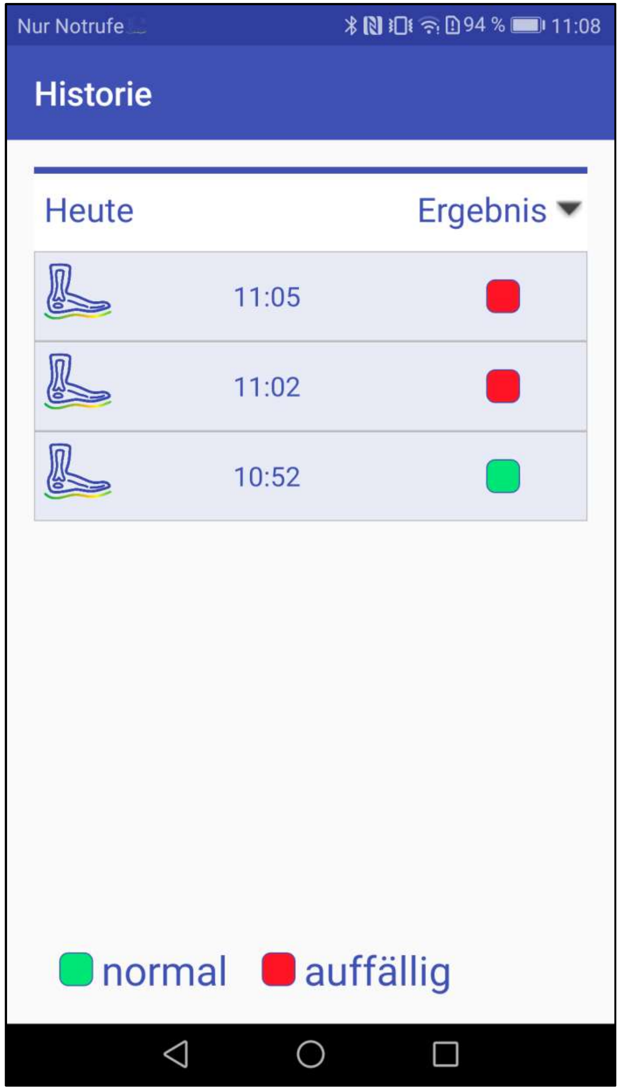

to view the measurement result

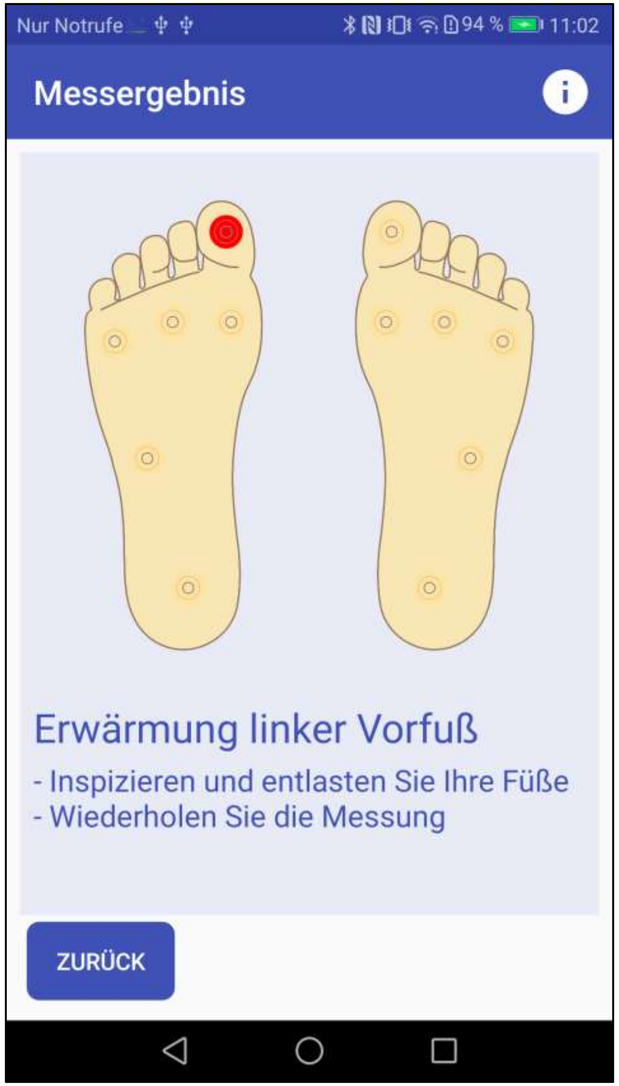

1. Rest your feet

mobile phone application

Result messages

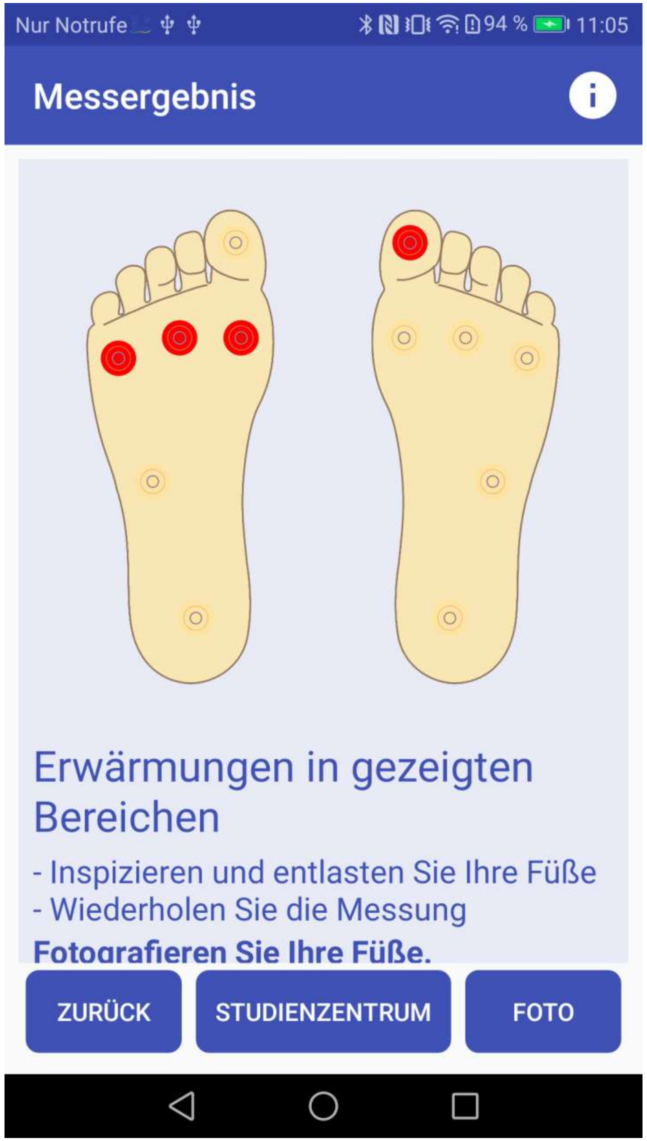

Result messages

mobile phone application

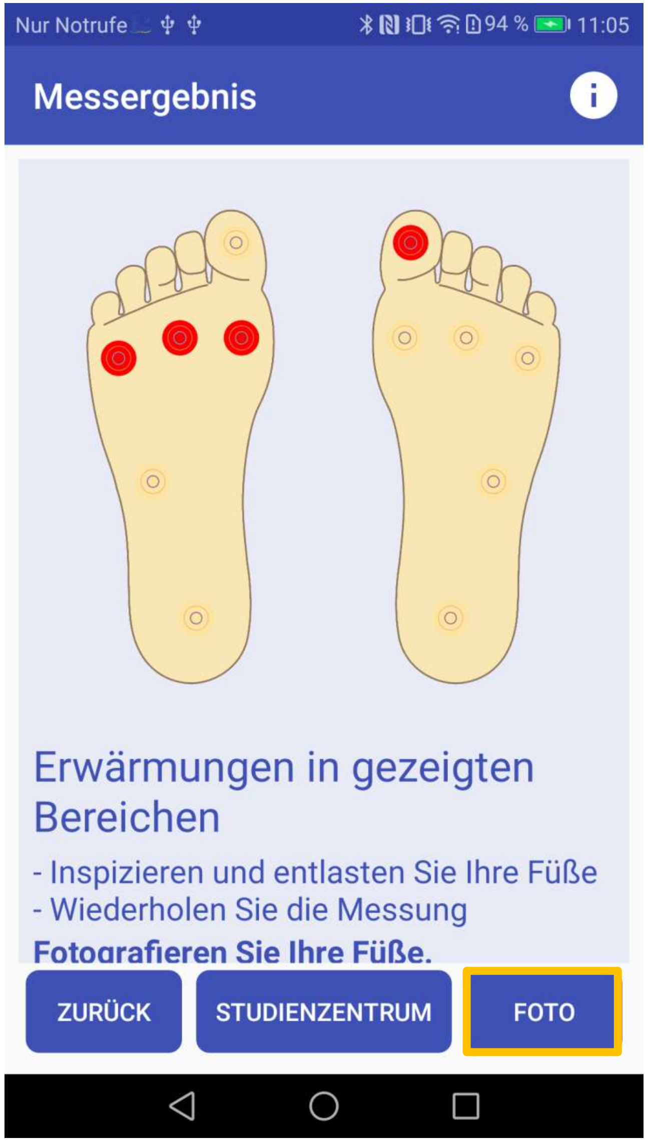

Evaluation of the scan - conspicuous findings

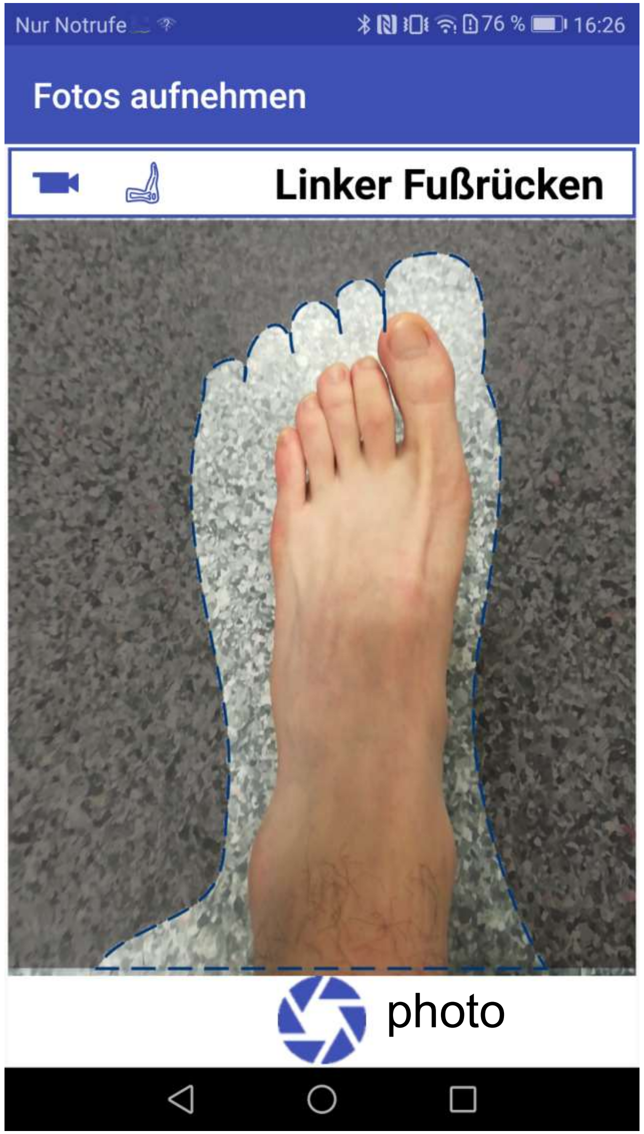

Photographing with camera of mobile phone

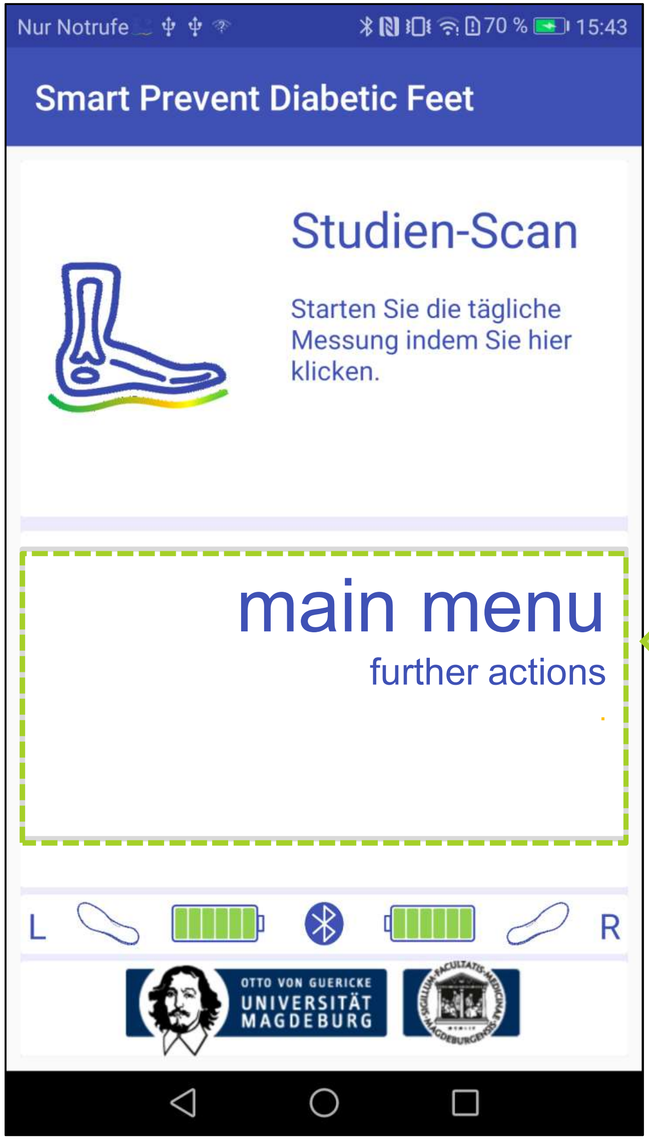

Start main menu

Photographing with camera of mobile phone

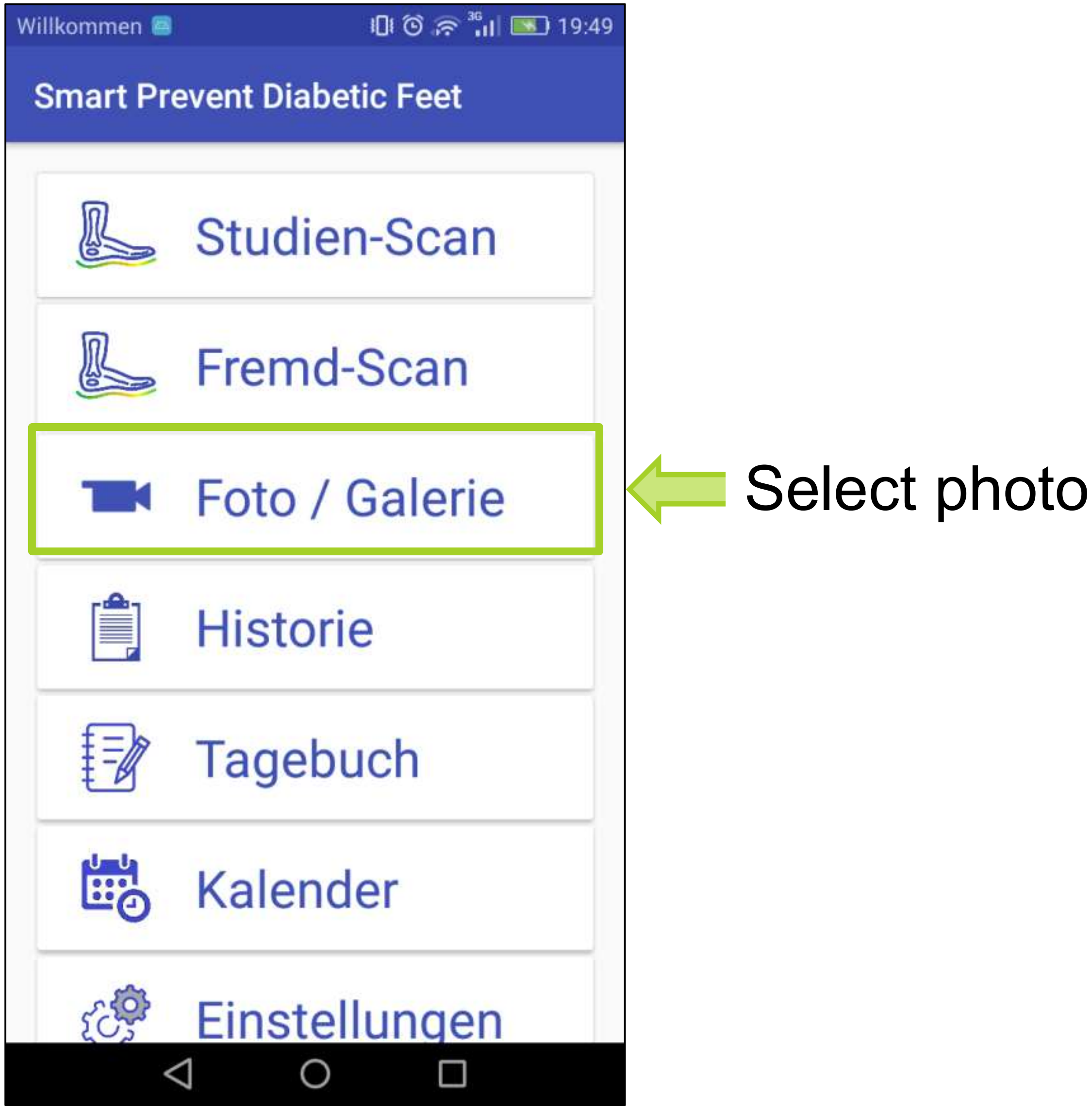

Photo / Gallery

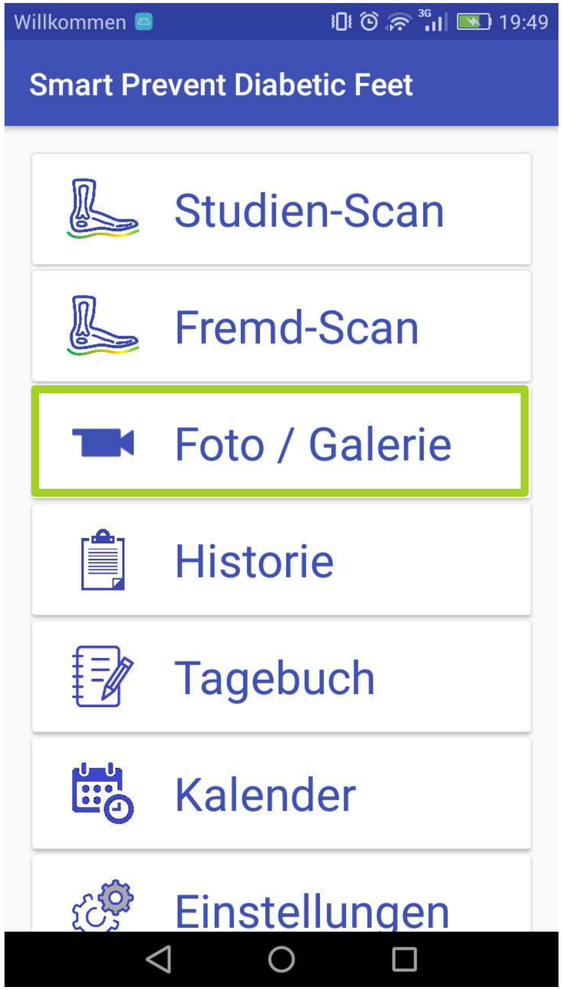

For **taking** photos when abnormality occurs to the foot (bladder)

&

Viewing photos

mobile phone application

Photographing with camera of mobile phone

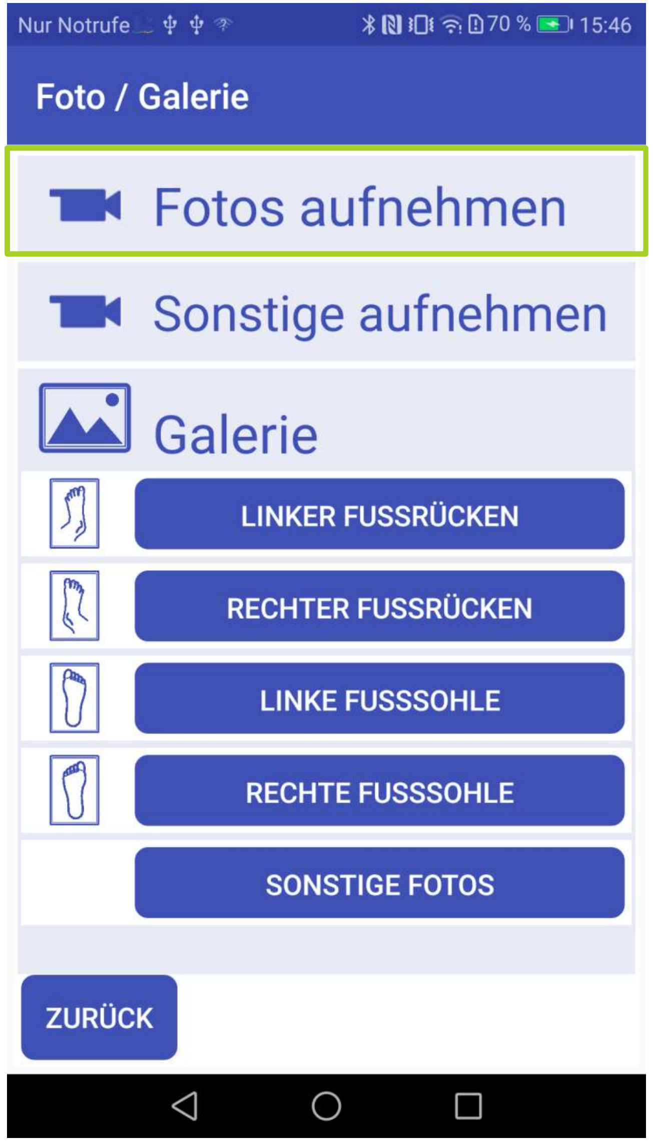

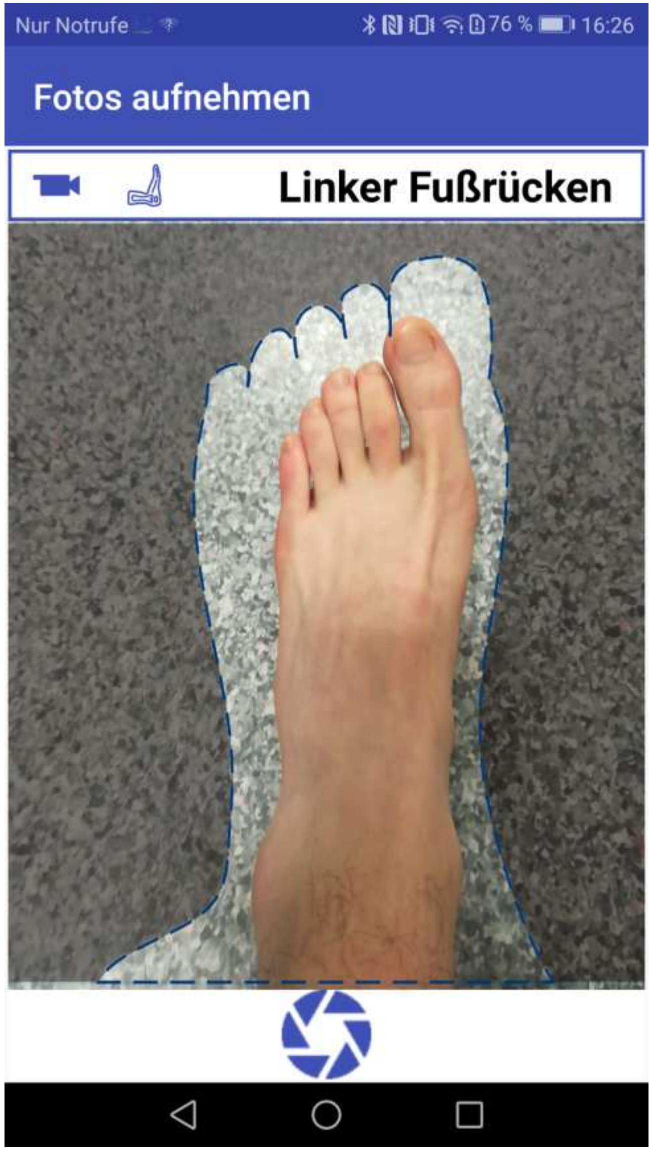

Photographing with camera of mobile phone

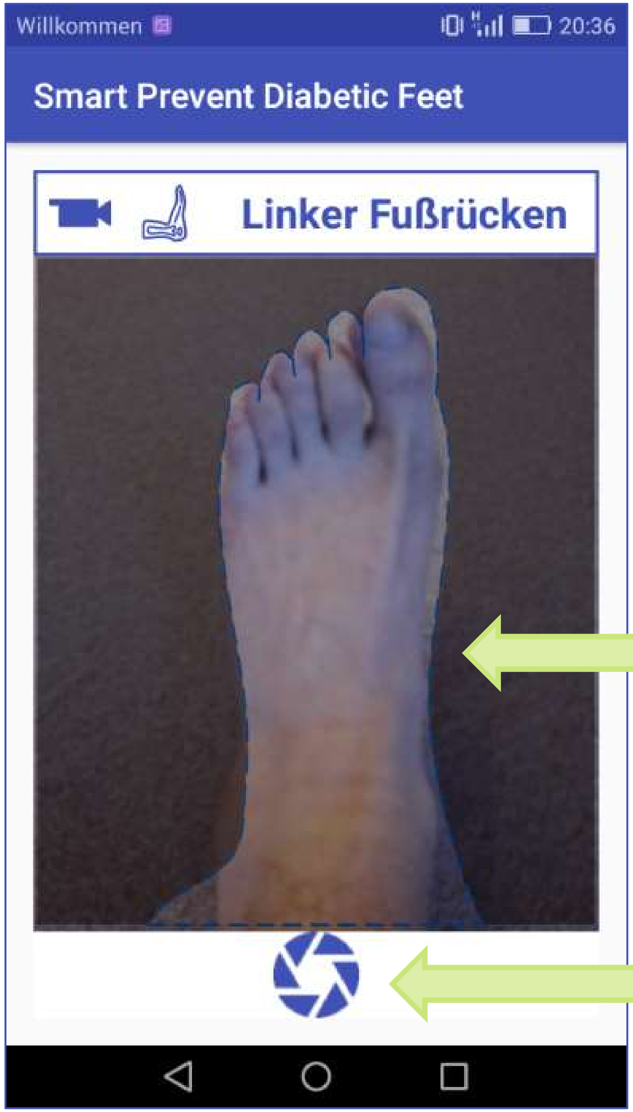

foot to be photographed

framework specification

capture

Photographing with camera of mobile phone

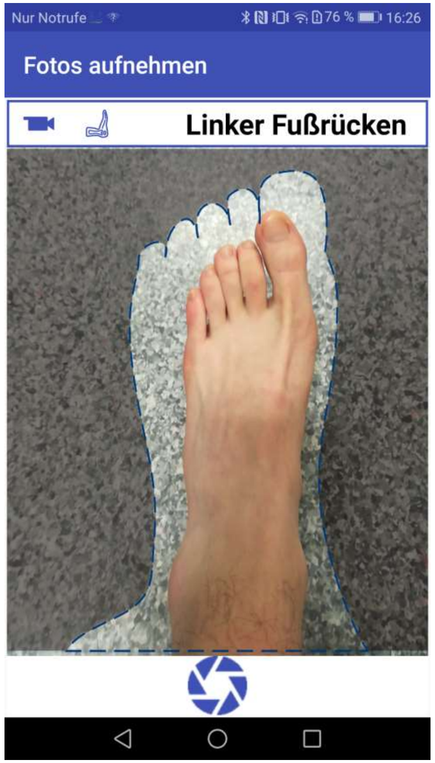

<u>Step one:</u>
Photo left arch of the foot

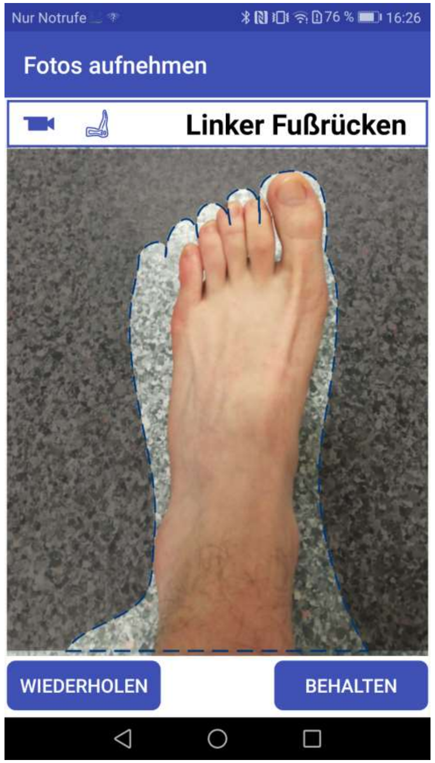

<u>Step two:</u>
Keep or discard photo

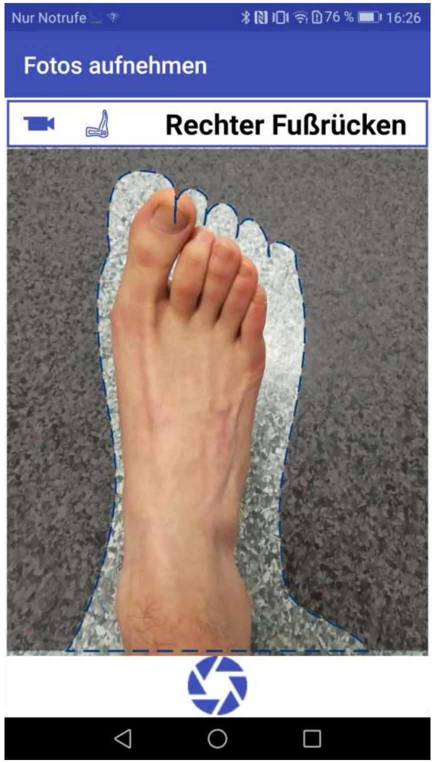

<u>Step three:</u>
Photo right back of foot

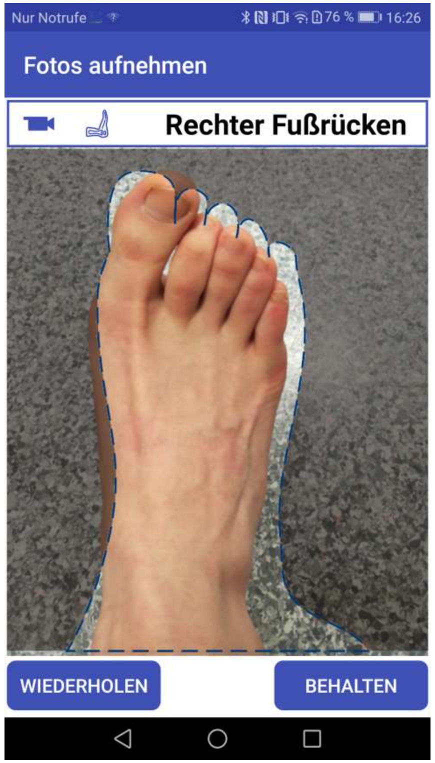

<u>Step four:</u>
Keep or discard photo

Photographing with camera of mobile phone

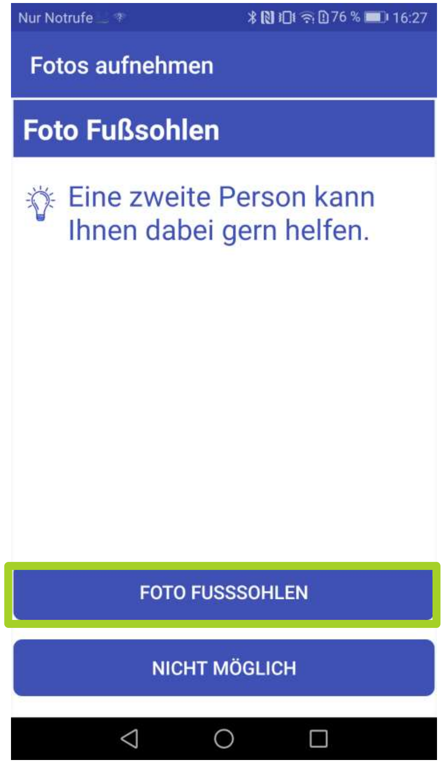

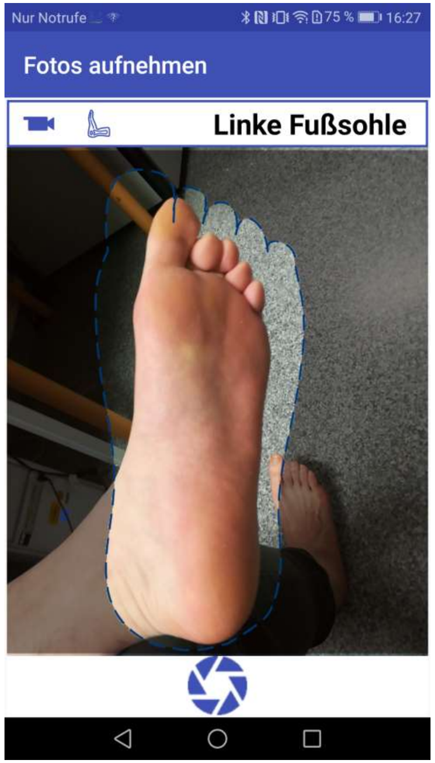

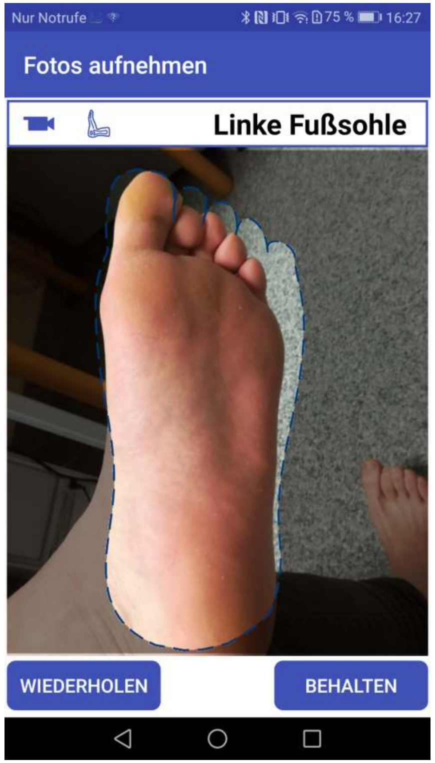

Step five:
Read the text of the note on soles

Step six:
Photo left sole of foot

Step seven:
Keep or discard photo

Photographing with camera of mobile phone

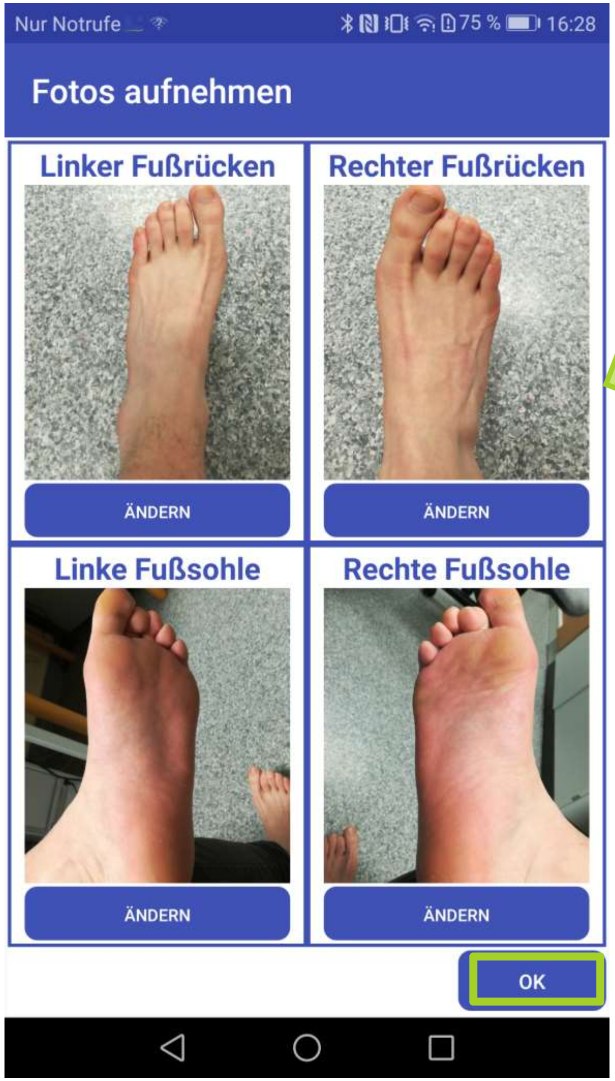

Repeat single images

Press Ok

mobile phone application

Photo / Gallery

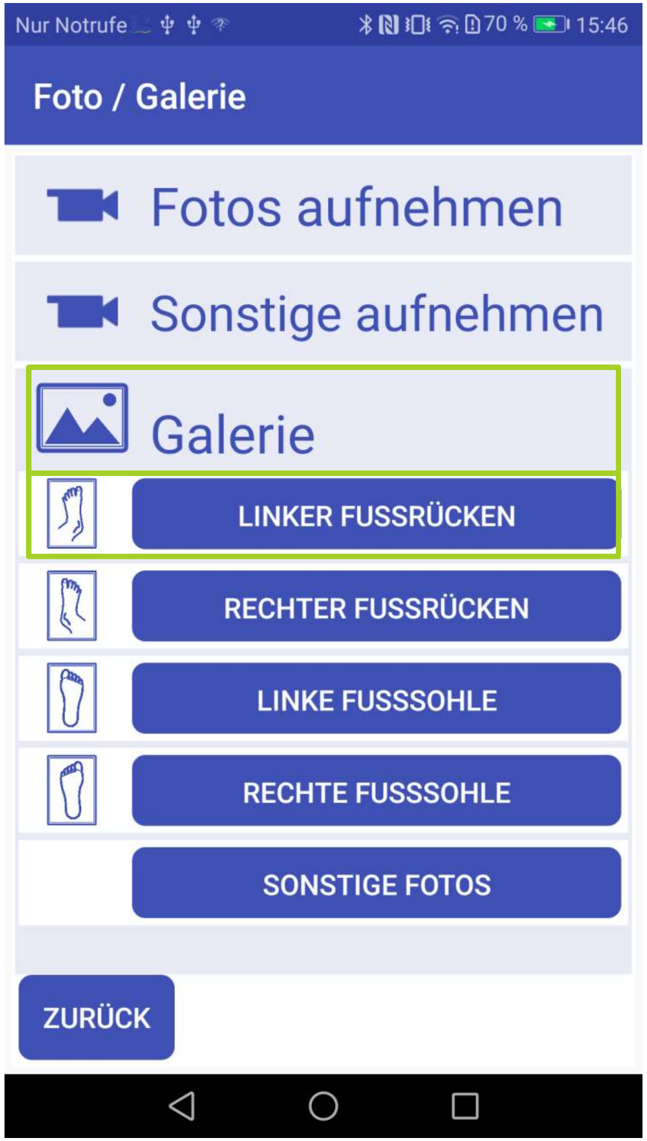

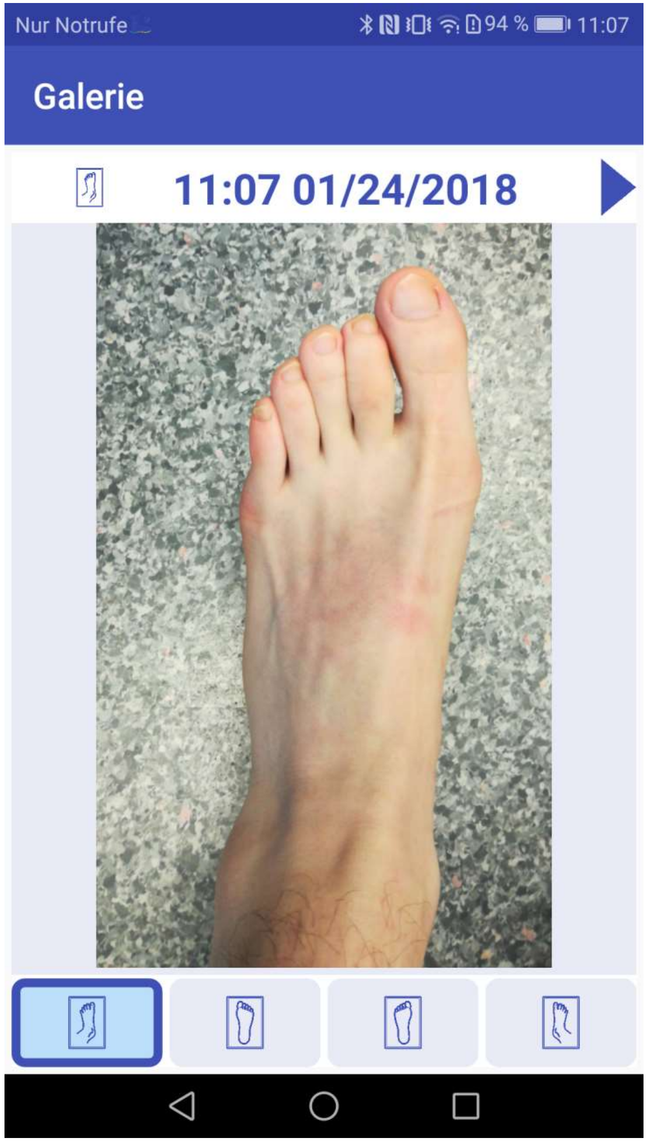

Photo / Gallery

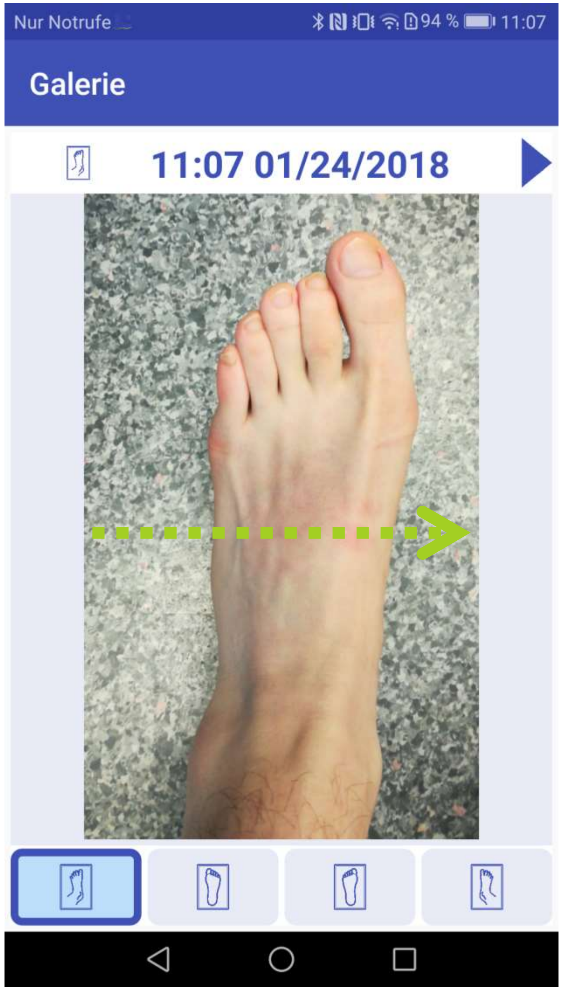

Date of recording

Wipe photos to the side with your finger to view them

Select foot areas

Create a diary entry

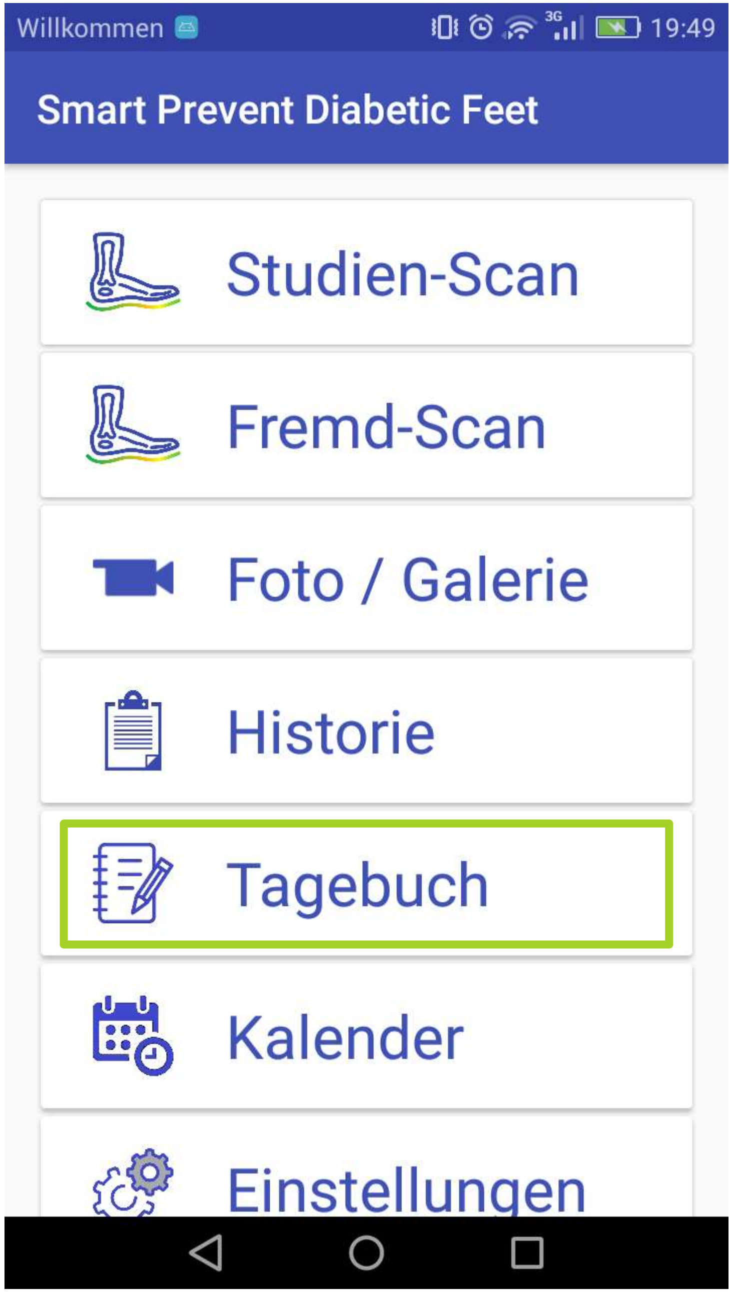

Notes on your **state of health** and daily foot examination

Home Diary

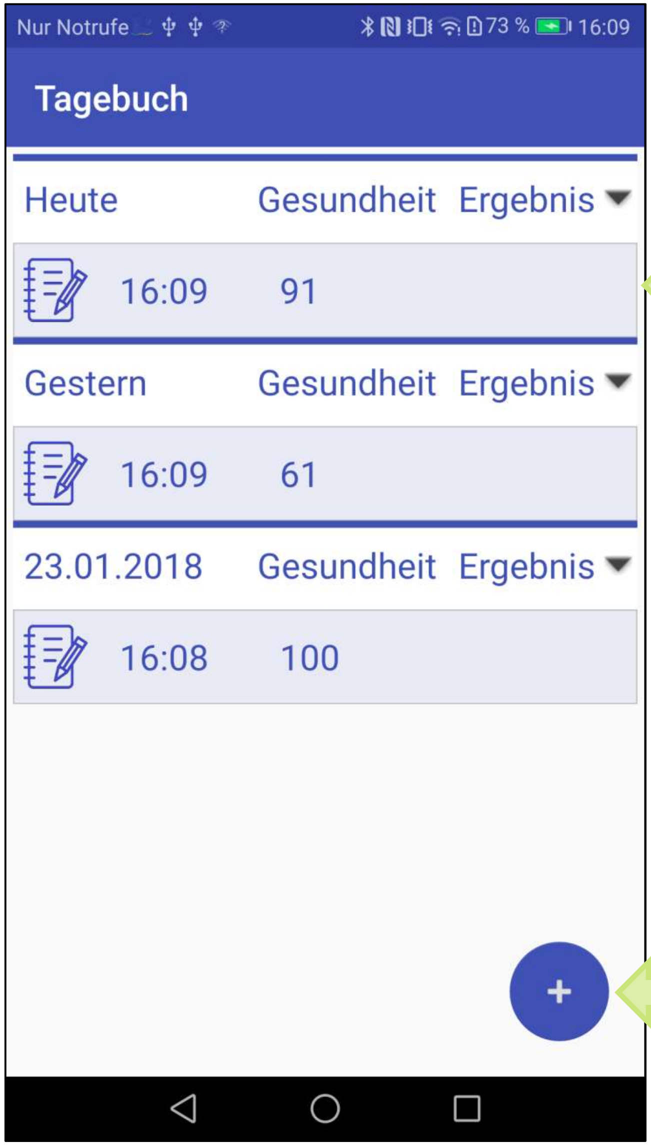

View / change entry

new entry

mobile phone application

New diary entry

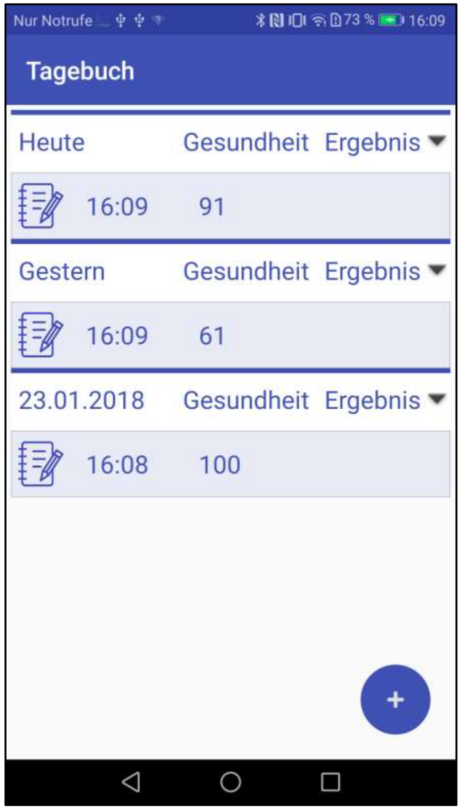

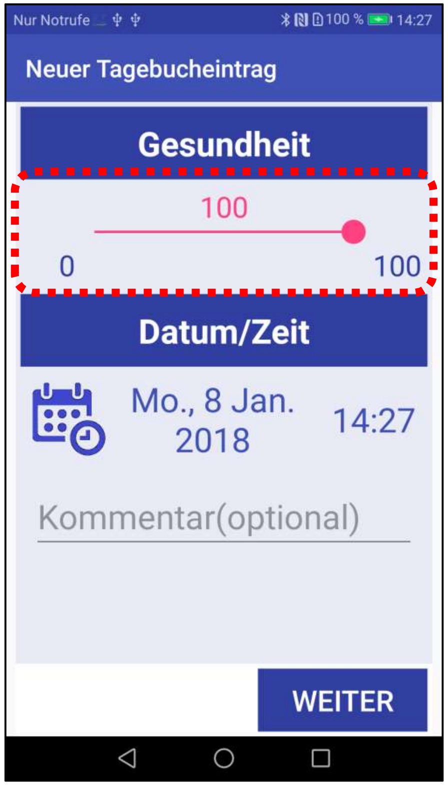

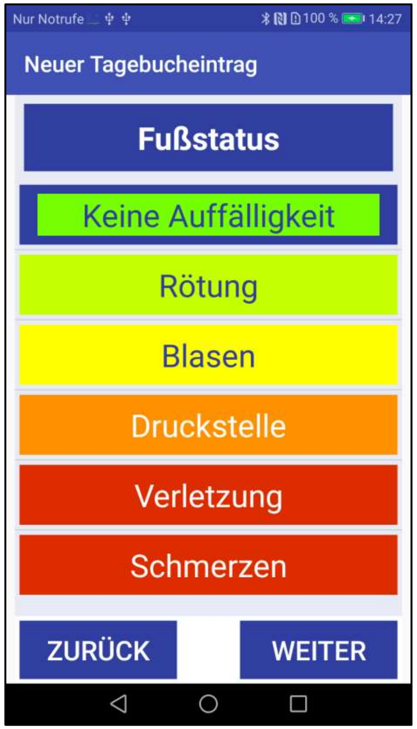

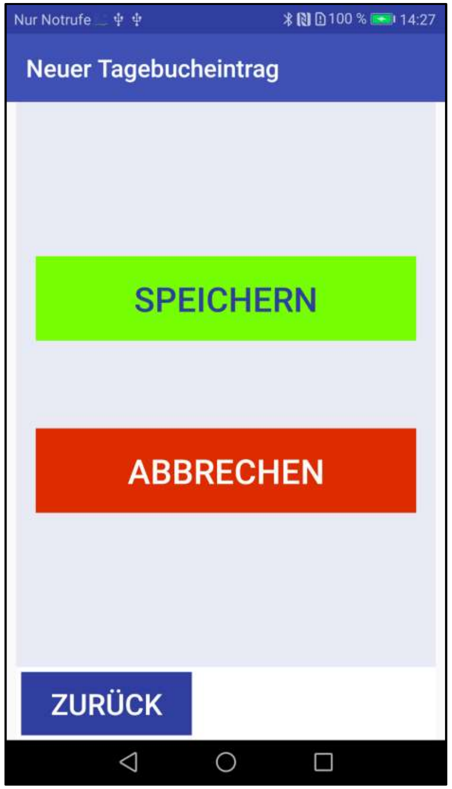

mobile phone application

Calendar / Dates

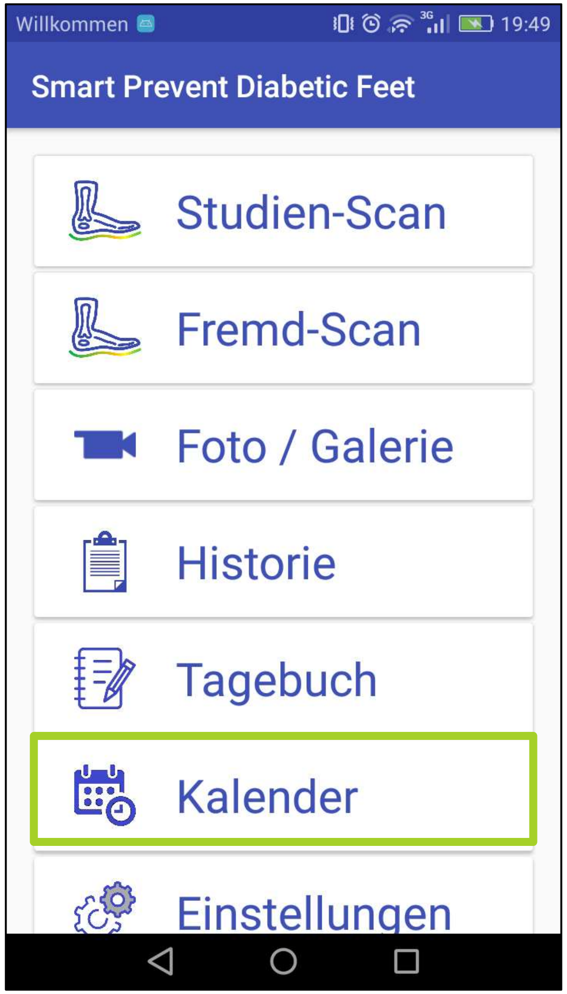

scheduling

Study visits, measurements,
medical appointments, taking medication, etc.

Calendar / Dates

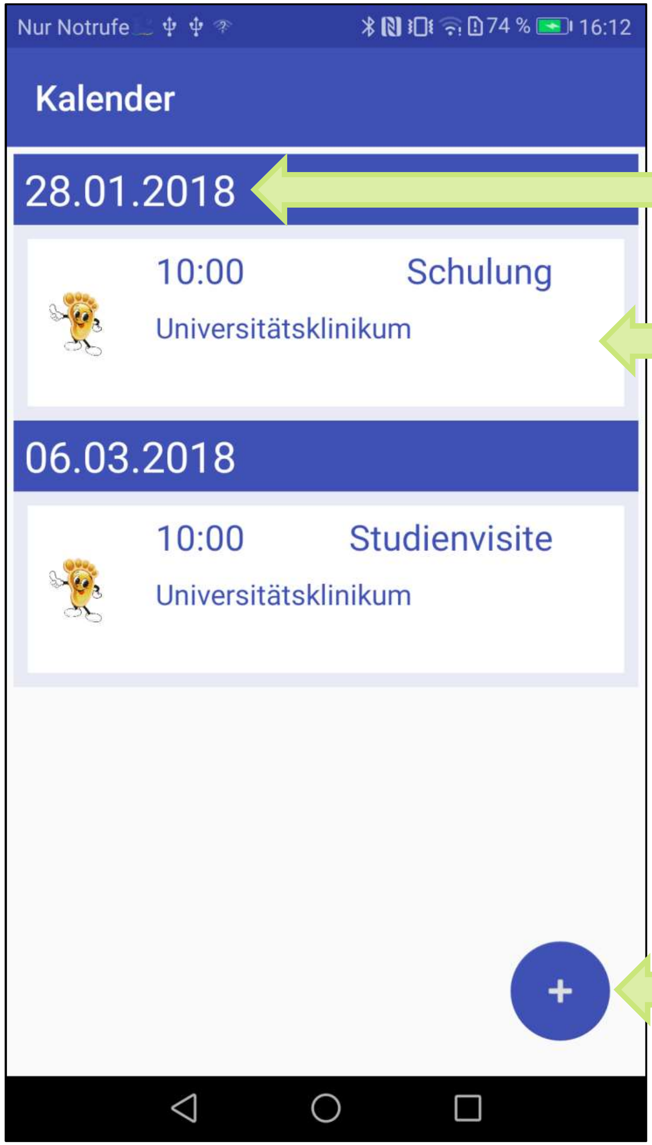

date

Date (time / type / location)

new appointment

New calendar entry

mobile phone application

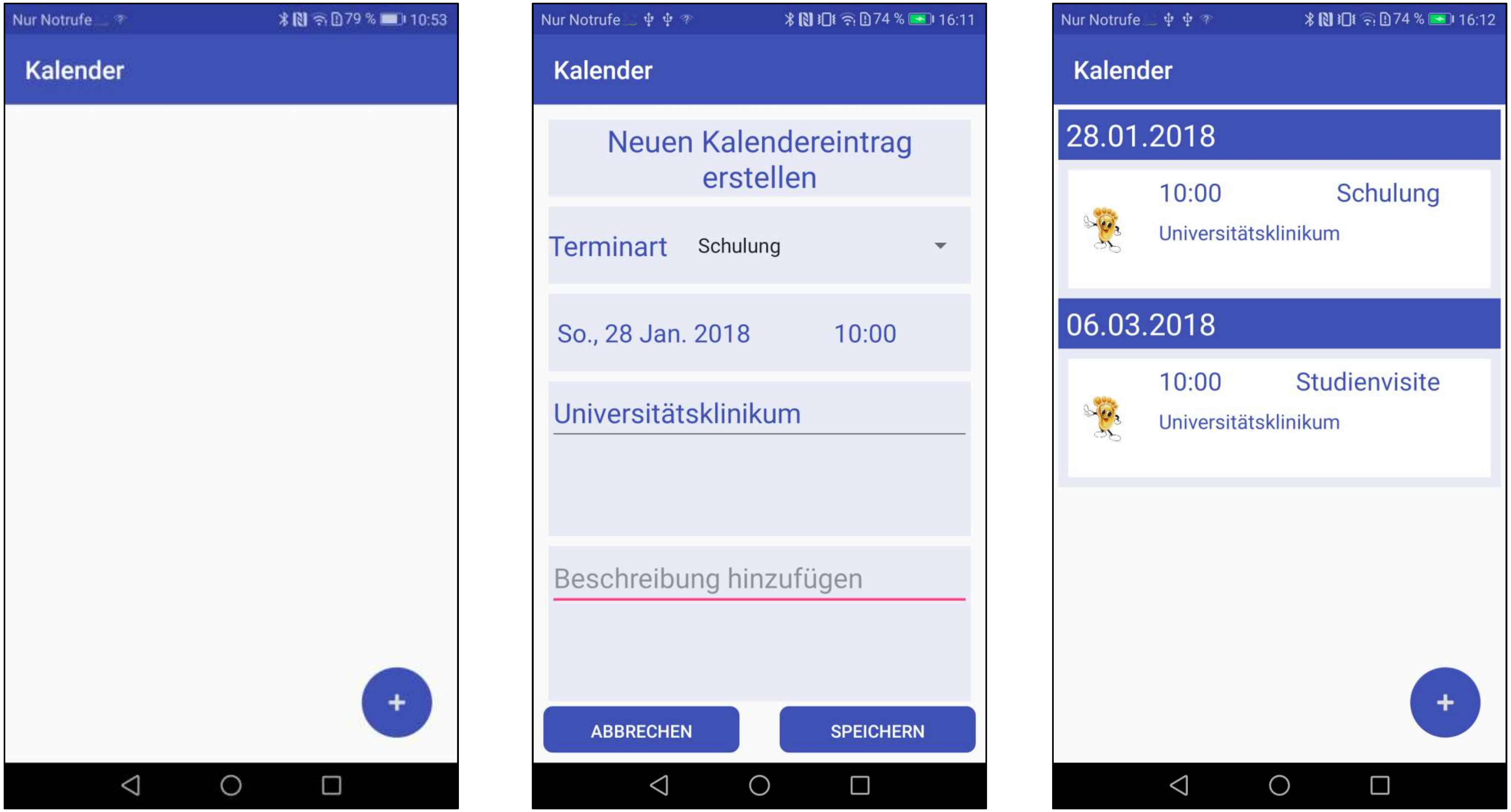

settings

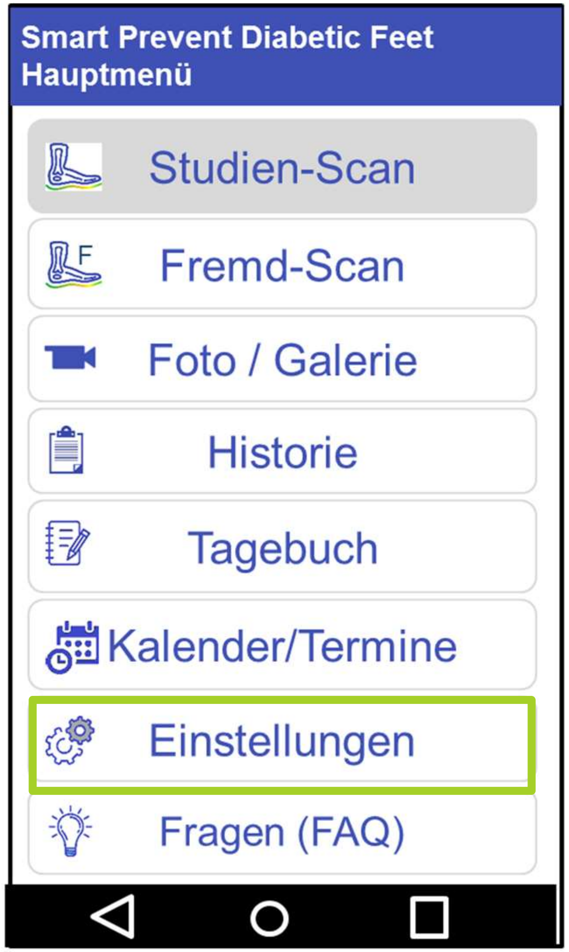

Hauptmenü

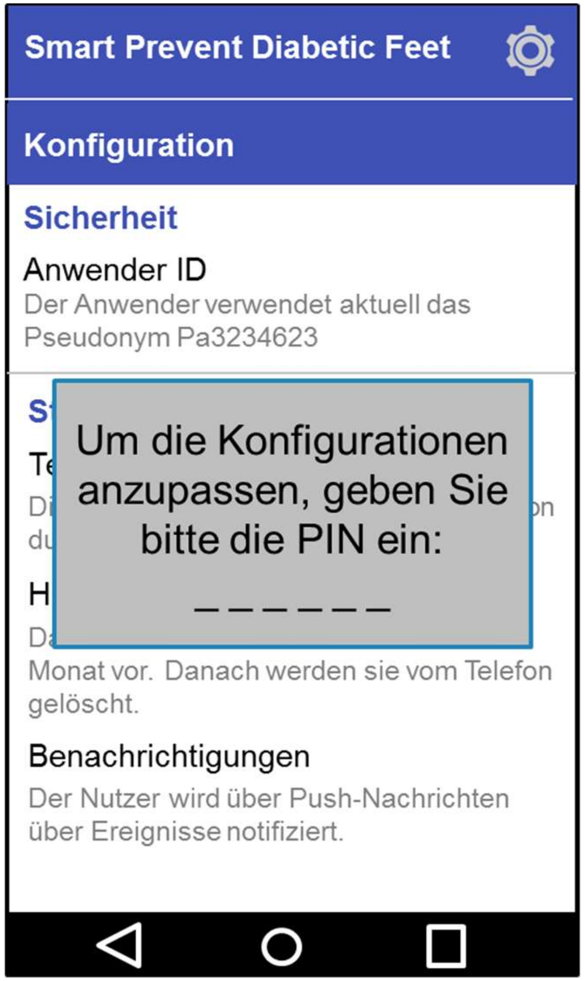

Only for
Study Center!!!

Questions (FAQ)

mobile phone application

Smart Prevent Diabetic Feet Questions (FAQ)

- Survey
- Battery
- Bluetooth and connection to the sole
- Intelligent insole
- Measurement
- Around the diabetic foot
- Sensors
- Software copyright notice
- Contact us

Smart Prevent Diabetic Feet Questions (FAQ)

- Sensors
- Software copyright notice
- Contact us

study leader

Prof. Dr. med. Peter R. Mertens
Director of the Clinic for Kidney and Hypertension Diseases, Diabetology and Endocrinology
Secretary's office: Mrs. C. Kluge
phone: 0391-6713236
Telefax: 0391-6715440

E-mail: Nephrologie@med.ovgu.de

study team

Coordinating Inspector

...and on vacation

take your shoes with you on holiday

Holidays in **Germany**: no restrictions

Holidays **abroad**: data transmission to the study centre is interrupted

Measured values are stored in the soles.

Transmission/evaluation on return

Feedback from the study centre only possible after returning home

In case of longer planned measurement failures, **please inform Study Centre**

... in case of illness

For inpatient stays and planned measurement failures please contact Study Center (by phone)

Functions of the App

Detect and prevent ulcers
study scan

Document foot injuries
Photo/Gallery

Keeping an eye on your well-being
diary

For technical problems

Reasons for incorrect measurements

1. soles are not sufficiently loaded
2. other devices interfere with reception
3. observe the sequence of the measurement

Thank you for your attention!

Isabell.walter@med.ovgu.de
ahmad.alhajjar@med.ovgu.de
Clinic for Nephrology and Hypertension, Diabetology and Endocrinology
Otto von Guericke University Magdeburg

external scan

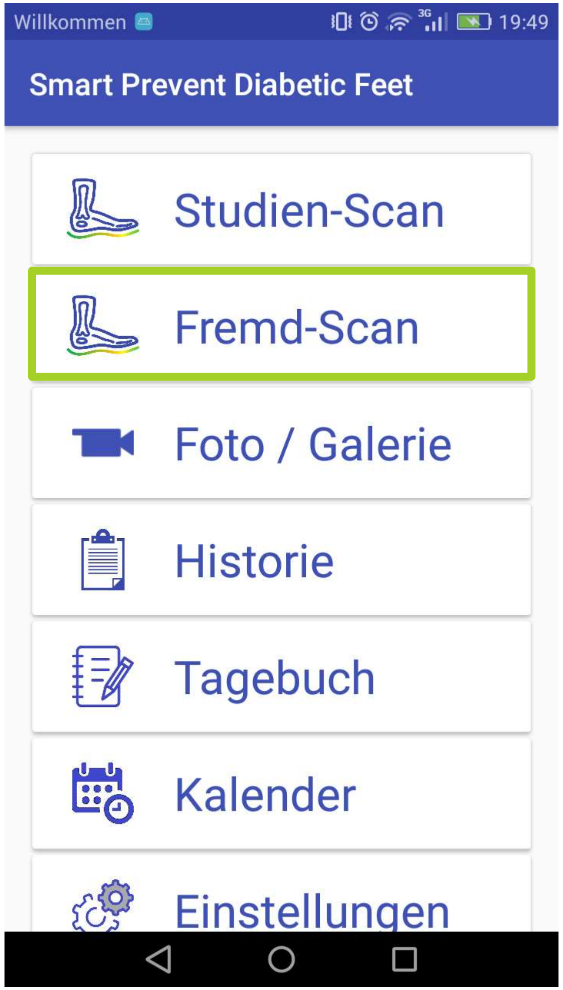

For friends & family to test the sole

Study scan without consequences (Values are not recorded in the study center)

Contact details

Clinic for Nephrology and Hypertension,
Diabetology and Endocrinology,
Otto-von-Gueircke University Magdeburg

House 60 B, Level 2

Phone: 0391 /67-21615

0391 /67-21745

E-mail: Claudia.Piehler@med.ovgu.de

Isabell.Walter@med.ovgu.de

Ahmad.Alhajjar@med.ovgu.de

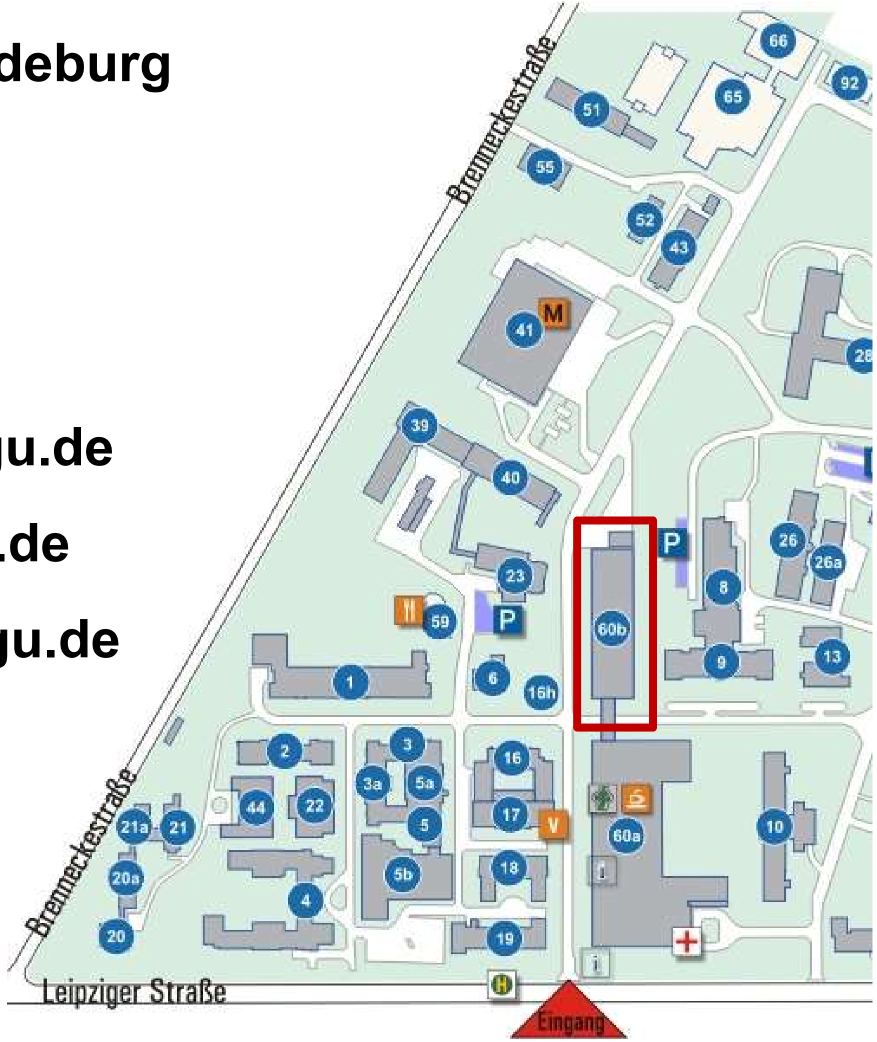

The study's app: recognition logo

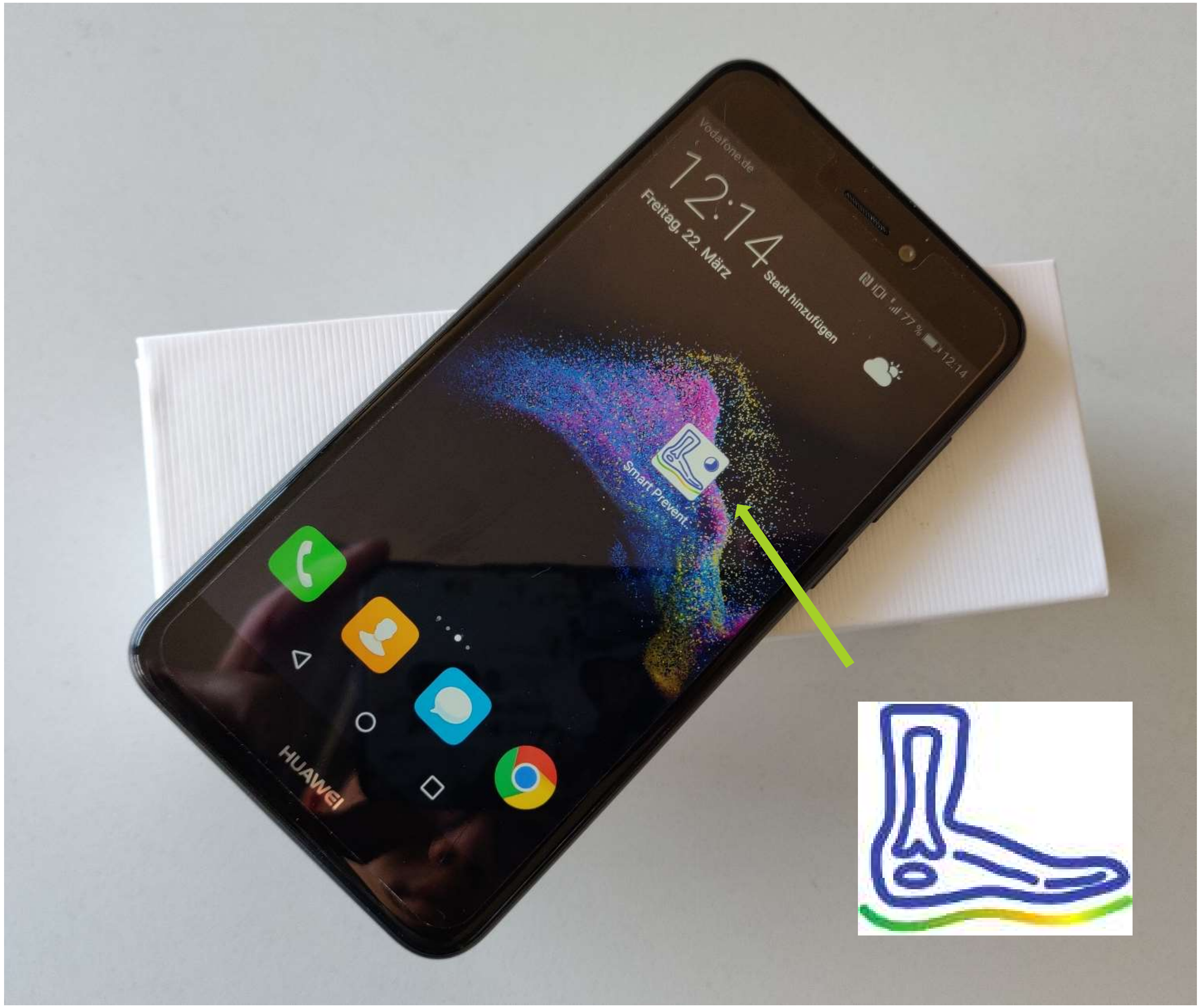

mobile phone application

study scan
